# Macrophages Break Interneuromast Cell Quiescence by Intervening in the Inhibition of Schwann Cells in the Zebrafish Lateral Line

**DOI:** 10.3389/fcell.2022.907863

**Published:** 2022-07-01

**Authors:** Meng-Ju Lin, Chia-Ming Lee, Wei-Lin Hsu, Bi-Chang Chen, Shyh-Jye Lee

**Affiliations:** ^1^ Department of Life Science, National Taiwan University, Taipei, Taiwan, R.O.C.; ^2^ Research Center for Applied Sciences, Academia Sinica, Taipei, Taiwan, R.O.C.; ^3^ Research Center for Developmental Biology and Regenerative Medicine, National Taiwan University, Taipei, Taiwan, R.O.C.; ^4^ Center for Biotechnology, National Taiwan University, Taipei, Taiwan, R.O.C.

**Keywords:** regeneration, lateral line, interneuromast cell, Schwann cell, Wnt, macrophage

## Abstract

In the zebrafish lateral line system, interneuromast cells (INCs) between neuromasts are kept quiescent by underlying Schwann cells (SWCs). Upon severe injuries that cause the complete loss of an entire neuromast, INCs can occasionally differentiate into neuromasts but how they escape from the inhibition by SWCs is still unclear. Using a genetic/chemical method to ablate a neuromast precisely, we found that a small portion of larvae can regenerate a new neuromast. However, the residual regeneration capacity was hindered by inhibiting macrophages. Using in toto imaging, we further discovered heterogeneities in macrophage behavior and distribution along the lateral line. We witnessed the crawling of macrophages between the injured lateral line and SWCs during regeneration and between the second primordium and the first mature lateral line during development. It implies that macrophages may physically alleviate the nerve inhibition to break the dormancy of INCs during regeneration and development in the zebrafish lateral line.

## Introduction

Tissue regeneration is critical for maintaining homeostasis and restoring organ function after injury in multicellular organisms. Planarians can almost replenish the whole organism from neoblasts (Newmark and Sánchez Alvarado, 2000; Wagner et al., 2011). In contrast, mammals only retain a limited regeneration capacity to replace damaged tissues. Injuries cause cell loss and damage to organs and tissues. Scars form in mammals to prevent further loss of cells and are often associated with a poor regeneration response. In addition, the destruction of mechanosensory hair cells within the inner ear by antibiotics, noise, or aging may cause a hearing deficit. However, hair cells could be recovered after damage in lower vertebrates such as birds, amphibians, and fish (Williams and Holder, 2000; Bermingham-McDonogh and Rubel, 2003; Stone and Cotanche, 2007; Hernandez et al., 2011; Romero-Carvajal et al., 2015). Intriguingly, mammalian cochlea multipotent progenitor cells could be cultured *in-vitro* under some conditions. Those cells could potentially complement the hair cell loss (Li et al., 2003; Sinkkonen et al., 2011; Shi et al., 2012; Jan et al., 2013). It suggests that genetic machinery for tissue renewal is still there in most animal species but not the regulatory mechanisms required to awaken the dormant multipotent progenitor cells (Liu et al., 2013; Kang et al., 2016). What are the regulatory factors? How is the responsiveness of progenitor cells regulated at the cellular and molecular levels? How may the degree of stimuli result and regulate differential responsiveness of potential progenitor cells? To investigate these questions, we study zebrafish (*Danio rerio*), a well-established vertebrate model with a remarkable regeneration capacity in most tissues and organs, including the lateral line system.

**FIGURE 1:**
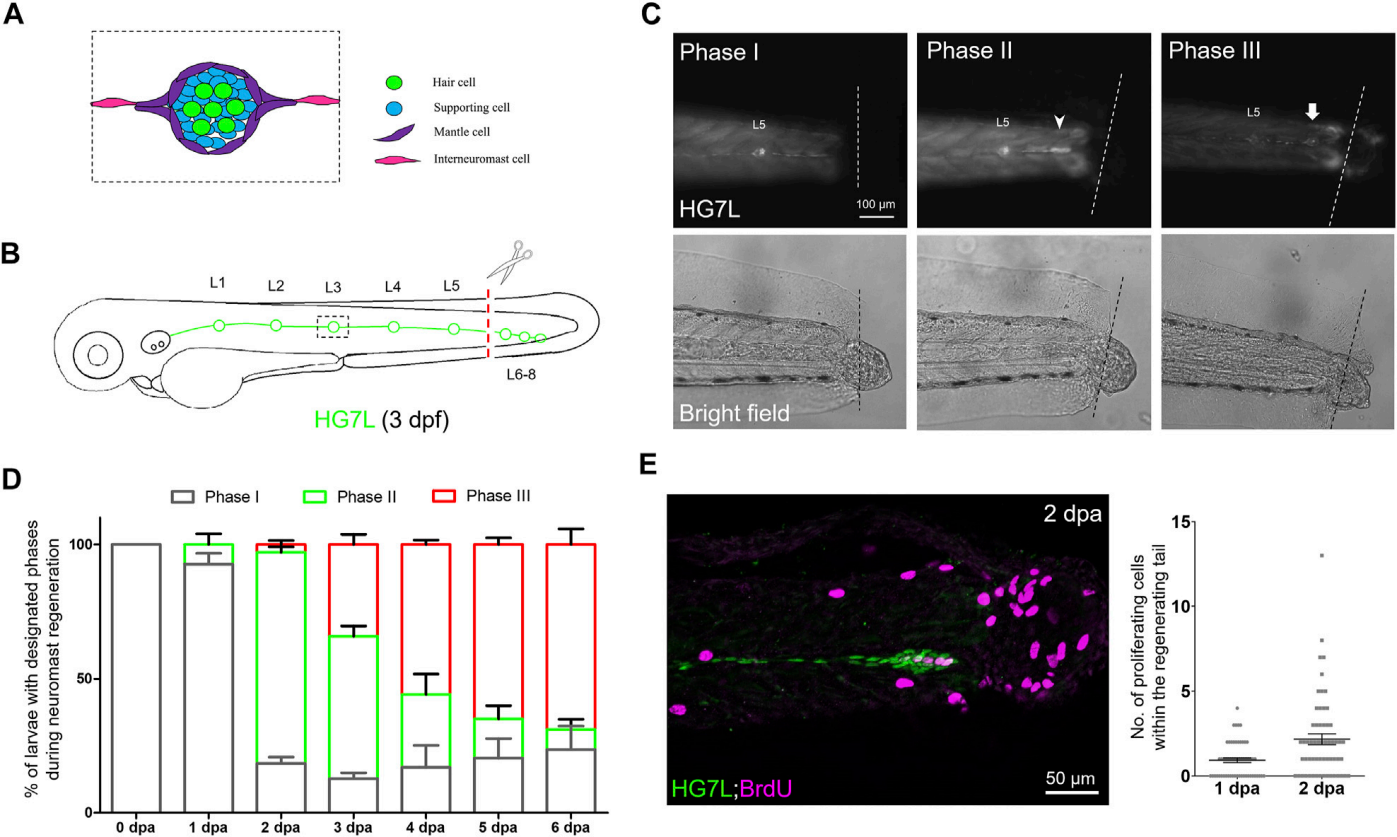
Active cell proliferation and clustering occur during neuromast regeneration upon fin amputation. **(A)** A cartoon shows different cell types in a neuromast of the lateral line. **(B)** A graph shows only one side of the fluorescent posterior lateral (green) with L1-8 neuromasts of an *Et(HG7L)* larva at three days post-fertilization. The tail fin is clipped at the dashed line to remove neuromast L6-8. **(C)** A new neuromast was regenerated in three distinct phases as examined at the GFP channel under epifluorescent microscopy. Phase I: No notable increase in fluorescent cells was observed in the lateral line between the L5 neuromast (as labeled) and the cut site (dotted line). Phase II: Fluorescent cells were increased and aggregated to form a cluster (arrowhead). Phase III: A new neuromast was formed (arrow). The corresponding bright-field images for each phase are shown below. **(D)** The percentages of larvae at each phase were calculated on the designated day postamputation (dpa, N = 3, *n* = 70). **(E)** *Et(HG7L)* larvae were fin-amputated, fixed at 1 and 2 dpa, and subjected to BrdU or EdU staining (in magenta) to probe actively proliferating cells or GFP immunohistochemistry (in green) to stain lateral line. Active cell proliferation was observed near the cutting edge at 2 dpa and quantified in a scatter plot on the right.

The lateral line system is a mechanosensory system that detects water movements around fish bodies, contributing to navigation, schooling, and predator avoidance (Montgomery et al., 1997; Stewart et al., 2013). The zebrafish lateral line system includes anterior and posterior-lateral lines flanking both lateral sides of the fish body. The sensory organs of the lateral line system are neuromasts. During development, neuromasts are periodically deposited during the posterior migration of a posterior lateral line primordium (pLLp), a migrating cluster of about a hundred cells (Nogare et al., 2017). A neuromast is composed of centrally-positioned hair cells, which are functionally and structurally similar to the hair cells of the mammalian inner ear. Hair cells are protected by supporting cells (SCs) or mantle cells (MCs), which are progenitor cells during hair regeneration (Figure 1A) (Ghysen and Dambly-Chaudière, 2007; Chitnis et al., 2012). The lateral line hair cells are exposed on the body surface and constantly face mechanical and chemical environmental assaults. One can efficiently ablate lateral line hair cells by exposing zebrafish larvae to heavy ions such as copper (Hernández et al., 2006; Olivari et al., 2008; Hernandez et al., 2011), mercury (Liang et al., 2003), or antibiotics such as neomycin (Harris et al., 2003). A previous study reported that SCs underneath hair cells are notable progenitor cells compared to the center or anterior differentiating or dormant support cells (Romero-Carvajal et al., 2015). In addition, MCs encircling the neuromast can re-enter the S phase to form a new neuromast facing a high copper ion concentration (Romero-Carvajal et al., 2015) and a severe injury such as tail fin amputation (Dufourcq et al., 2006; Wada et al., 2013). Lastly, interneuromast cells (INCs) sitting in between neuromasts are kept quiescent by the underlying Schwann cells (SWCs) and posterior lateral line nerve (pLLn) connecting to hair cells. Perturbation of the epidermal growth factor (EGF) pathway between SWCs and pLLn results in early activation of INCs and precocious intercalary neuromast formation (López-Schier and Hudspeth, 2005; Lyons et al., 2005; Perlin et al., 2011; Lush and Piotrowski, 2014). During the migration of the 2^nd^ pLLp, the intercalary neuromast formation also occurs by blocking the contact between INCs and SWCs (Ledent, 2002; Ghysen and Dambly-Chaudière, 2007; Nuñez et al., 2009). A new neuromast regenerates after the electro-ablation of a whole neuromast (Sánchez et al., 2016). These studies nicely demonstrate the regeneration capacity of INCs. By RNA-seq analysis and functional assay, MCs and INCs have been shown to contribute to hair cell regeneration by interrogating multiple signaling pathways such as Notch, Wnts, Fgfs, and retinoic acid sequentially and spatially (Jiang et al., 2014; Steiner et al., 2014). However, it is still puzzling to understand the cellular hierarchy of lateral line regeneration while facing diversified external cues.

Upon damage, macrophages infiltrate into the tissue to promote regeneration (Stefater et al., 2011; Keightley et al., 2014). Macrophages acquire polarity transition from the M1-like macrophages, classically regarded as activated pro-inflammatory cells, to the M2-like phenotype known as alternatively activated macrophages involved in inflammation resolution (Sica and Mantovani, 2012; Nguyen-Chi et al., 2015). Macrophages clean up infected neutrophils, debris, and dying cells by phagocytosis (Martin et al., 2014) and secret anti-inflammatory cytokines such as IL-10 and TGFβ to suppress the chronic inflammatory response (Chazaud, 2014; Wynn and Vannella, 2016). Recent data suggest that macrophages enhance regeneration by secreting cytokines like TNFα and VEGF. TNFα is critical for blastemal formation during fin regeneration in zebrafish (Nguyen-Chi et al., 2017). VEGF helps angiogenesis, wound repair, and renewal of blood vessels and peripheral nerves (Fantin et al., 2010; Cattin et al., 2015; Gurevich et al., 2018). Macrophages could also go through canonical or non-canonical Wnt pathways to influence regeneration (Lin et al., 2010; Stefater III et al., 2011). In addition to cytokine effects, macrophages could exert mechanical forces to facilitate wound repair and regeneration (Liu et al., 2016; Gurevich et al., 2018). As expected, macrophages play a pivotal role in hair cell regeneration compared to neutrophils (Carrillo et al., 2016). However, how macrophages facilitate lateral line regeneration upon tissue damage remains unclear.

In this work, we first provide further evidence to support that INCs are the primary progenitor cell source to replenish an entire neuromast post the amputation of the tail fin. To our surprise, a few larvae could regenerate a neuromast after ablation without harming SWCs and pLLn. By using *in-toto* imaging, we found macrophages are significantly patrolling damaged sites. In addition, we also observed that macrophages squeeze in between INCs and SWCs/pLLn. It suggests that macrophages may break INC quiescence by intervening in the inhibition of SWCs in the zebrafish lateral line system.

## Results

### Regeneration of Neuromast Post Fin Amputation

Few studies address the regeneration of an entire lateral line neuromast in zebrafish. Here, we generated an enhancer-trap line, *Et(HG7L)*, expressing enhanced green fluorescent protein (EGFP) in both MCs and INCs in the lateral line (Supplementary Figure S1). By sequencing, we found that the Tol2 construct was inserted in between *ccdc147* and *sorcs3*. At two-three days post-fertilization (dpf), the *Et(HG7L)* embryos also exhibit a high EGFP fluorescence in the head region (Supplementary Figure S1C), resembling the expression of *sorcs3* as examined by whole-mount *in situ* hybridization (WISH) (Supplementary Figures S1E and F). In addition, EGFP fluorescence was found in the trunk (Supplementary Figure S1D), similar to the expression of *ccdc147* shown by WISH (Supplementary Figure S1F).

At 3 dpf, the *Et(HG7L)* embryos show green fluorescence in the lateral line, including neuromasts (Supplementary Figures S1G and H). We acquired another transgenic line, *Tg(-4.7alpl:mCherry)*, showing mCherry in MCs and their associated INCs (Supplementary Figures S1I and J) (Steiner et al., 2014). Embryos from the crossing of two lines result in overlapping EGFP and mCherry signals in INCs and MCs but not SCs (Supplementary Figures S1K and L). To better visualize the difference in fluorescent labeling between two lines, a schematic drawing is presented in Figure S1M.

Using *Et(HG7L)* larvae at 3 dpf, we removed the distal neuromast cluster (L6-8) by amputating the caudal fin as indicated in Figure 1B under a stereo fluorescent microscope. We observed the change of fluorescent lateral lines at designated time points in bright and dark fields under an epifluorescent microscope. A significant portion of larvae grew a new neuromast near the wound site within days. We divided the entire process into three phases. In Phase I, no cell clusters in the injured lateral line; In Phase II, a cell cluster was found in the damaged lateral line; In Phase III, a new neuromast formed (Figure 1C). At one day post—fin amputation (dpa), most larvae (92.7%, *n* = 70) stayed in Phase I. However, they quickly shifted to Phase II in 78.7% of larvae at 2 dpa. The cluster formation might be due to active cell proliferation. Indeed, cell proliferation at the lateral line significantly increased as examined by BrdU or EdU labeling at 2 dpa compared to 1 dpa (Figure 1E). Even though no cell clustering was notable in Phase I, cell proliferation did occur near the cut site of the lateral line. It suggests that cells gradually pile up to form a cluster, as seen in Phase II.

A maturing neuromast forms a rosette-like structure in the center (Chitnis et al., 2012). Claudin B is a tight junction protein highly expressed in sensory organs, including the lateral line (Kollmar et al., 2001). We obtained a transgenic line, *Tg (−8.0cldnb:lyn-GFP)*, expressing membrane-bound EGFP in all neuromast cell types (Haas and Gilmour, 2006). By crossing it to the *Tg (−4.7alpl:mCherry)* line, we observed the formation of the rosette as evident with an intensified EGFP signal (Supplementary Figure S2C, arrowheads) in the center of a regenerating cluster of a fin-amputated larva (Supplementary Figure S2B) (Haas and Gilmour, 2006; Lecaudey et al., 2008). Tight junctions formed during apical cell assembly in the rosette (Chitnis et al., 2012). We, therefore, confirmed the existence of rosette by immunostaining against ZO-1, a tight junction-associated protein (Lecaudey et al., 2008), in 2-dpa *Et(HG7L)* larvae within the regenerating cluster (Supplementary Figure S2D). Interestingly, the cluster requires a larger area (1715.4 μm^2^, *n* = 10) and a smaller length/width ratio (6.79, *n* = 10) to accommodate one rosette, with its center mostly situated in the middle of the cluster (*n* = 16) (Supplementary Figure S2E). At 4 dpa, more than half of larvae (55.8%) had regenerated a new neuromast during observation.

Next, we examined the regeneration process more thoroughly under light-sheet fluorescence microscopy (LSFM), which is less photo-toxic and allows long-term recording (Power and Huisken, 2017). Multiple cells actively moved with protrusions (arrowheads) found in the leading end of the pLL system of larvae at both Phase I (Supplementary Video S1) and II stages (Supplementary Video S2). Unlike traditional collective migrations such as the pLLp migration during development, newly proliferated cells migrated toward injury sites in Phase I. The cells showed visible protrusions at the lagging end of the cluster in Phase II. Afterward, the homogenous cell cluster transformed into polarized cells with ring-like features of MCs within a new neuromast (Supplementary Video S3). These results suggest that a new neuromast is regenerated by active cell proliferation, clustering, and stochastic cell migrations.

### Interneuromast Cell is the Primary Cell Type Contributing to the Regeneration of Neuromast Post-Fin Amputation

**FIGURE 2:**
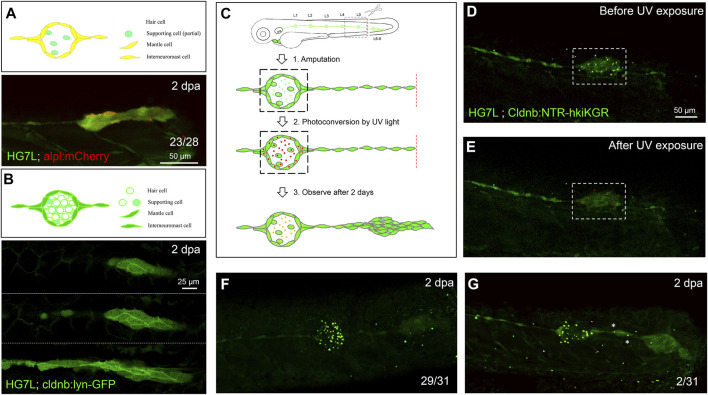
Interneuromast cells are the origin of clustering cells post–fin amputation. We performed fin amputation on double transgenic larvae from the cross of the *Et(HG7L)* with the *Tg(−4.7alpl:mCherry)* (alpl:mCherry) or *Tg (−8.0cldnb:lyn-GFP)* (cldnb:lynGFP) to track the origin of progenitor cells for the regenerating cluster post fin amputation at 2 days after amputation (2 dpa). **(A)** The regenerating cluster of fin-amputated larvae of *Et(HG7L) X Tg (−4.7alpl: mCherry)* line was examined at GFP or mCherry channel and photographed under confocal microscopy. A representative superimposed image from both channels is shown. The regenerating cluster image contains mostly yellow signals. As illustrated in the cartoon, the clustering cells could be originated from both interneuromast cells (INCs) and mantle cells (MCs). **(B)** The regenerating cluster of fin-amputated larvae of *Et(HG7L)* X *Tg (−8.0cldnb: lyn-GFP)* was examined at the GFP channel to scan the Z-axis. Images at three different z positions clearly show the existence of both membrane and cytosol green fluorescence. As illustrated in the cartoon, the signals could be originated from INCs, MCs, and supporting cells. **(C)** A series of cartoons shows utilizing a *Tg(−8.0cldnb:NTR-hKikGR;myl7:EGFP)* transgenic line, which expresses nitroreductase (NTR) and hKikGR fusion protein, to further discern whether the clustering cells are from INCs or cells inside a neuromast. To label the lateral line, we used larvae from the cross of *Et(HG7L)* (green) and *Tg(−8.0cldnb:NTR-hKikGR;myl7:EGFP)* (green dots), examined and photographed at the GFP channel before **(D)** and after UV exposure **(E)**. After UV exposure, the green *hKikGR* dots were converted to red in all cell types, including MCs within distal neuromast right after fin amputation. **(F)** At 2 dpa, labeled cells generated new green hKikGR proteins. As shown in **(F)**, the mingling of green and red signals becomes yellow. The labeled cells within the neuromasts rarely escaped in most treated larvae (29/31). However, we observed that a few larvae (2/31) showed yellow signals in the neighboring of INCs (white asterisks), as shown in **(G)**.

The loss of neuromast hair cells is known to be replenished by underlying SCs (Kniss et al., 2016), driven by differential Wnt or Notch signaling (Romero-Carvajal et al., 2015). In contrast, the roles of MCs and INCs are less appreciated. MCs seem to stay in quiescence unless facing severe damage (Romero-Carvajal et al., 2015). Given that different injury levels may arouse differential regeneration responses of the pLL system, we examined which type(s) of neuromast progenitor cell is/are the primary progenitor(s) for regenerating neuromasts post fin amputation. Since the *Et(HG7L)* line also expresses weak EGFP in a portion of SCs, we first used their larvae crossing with the *Tg (−4.7alpl:mCherry)* line with a dim mCherry (red) fluorescence found in MCs and INCs (Supplementary Figures S1I–M). The clusters showed overlapping signals at 2 dpa (Figure 2A). Moreover, we examined the regeneration of amputated larvae from the cross of the *Et(HG7L)* line and the *Tg(−8.0cldnb:lyn-GFP)* line and found that all the clustering cells showed both membrane and cytosol EGFP (Figure 2B). These results suggest that the clustering cells are mainly from MCs and INCs but not SCs.

Since none of the *in vivo* labeling methods or transgenic lines is available to distinguish MCs and INCs for their roles in neuromast regeneration, we established a double-transgenic line, *Tg(−8.0cldnb:NTR-hKikGR;myl7:EGFP)*, with a claudin-b promoter-driven expression of a fusion protein, of nitroreductase (NTR) (Jarrom et al., 2009) and a photo-convertible fluorescent protein (humanized Kikume Green–Red, abbreviated as hKikGR hereafter) (Tsutsui et al., 2005) in all cell types of the pLL system (see the gene construct in Supplementary Figure S3A). In addition, we also included a *myl7* promoter-driven EGFP gene in the transgene to express EGFP in the heart as a selection marker (see a schematic EGFP expression graph in Supplementary Figure S3A). Here, the hKikGR protein was expressed in punctate (Chen et al., 2011) in all cell types within neuromasts of the entire lateral line system, including the anterior and posterior lateral lines (Supplementary Figures S3B and C). The photo-convertible hKikGR protein can be activated to become red color to label the cells in the neuromast. We, thus, could examine whether cells in the neuromast can migrate out to differentiate into a new neuromast.

We amputated the tail fins of 3-dpf larvae from the cross of *Tg (−8.0cldnb:NTR-hKikGR)* and *Et (HG7L)* lines and then photo-converted hKikGR protein within the distal neuromast into red signals by ultraviolet light (Figures 2C–E, dashed rectangles). At 2 dpa, we found that red-fluorescent cells stay in the original neuromast in most larvae (Figure 2F). Only 2 out of 31 larvae had a few red-fluorescent cells outside the original neuromast (Figure 2G, asterisks). It further suggests that the INC, but not the SC nor the MC is the primary progenitor cell type to regenerate new neuromasts in the condition tested.

### Fin Amputation Diminishes Lateral Line Nerve Signal and Upregulates Wnt activity in Interneuromast Cells

**FIGURE 3:**
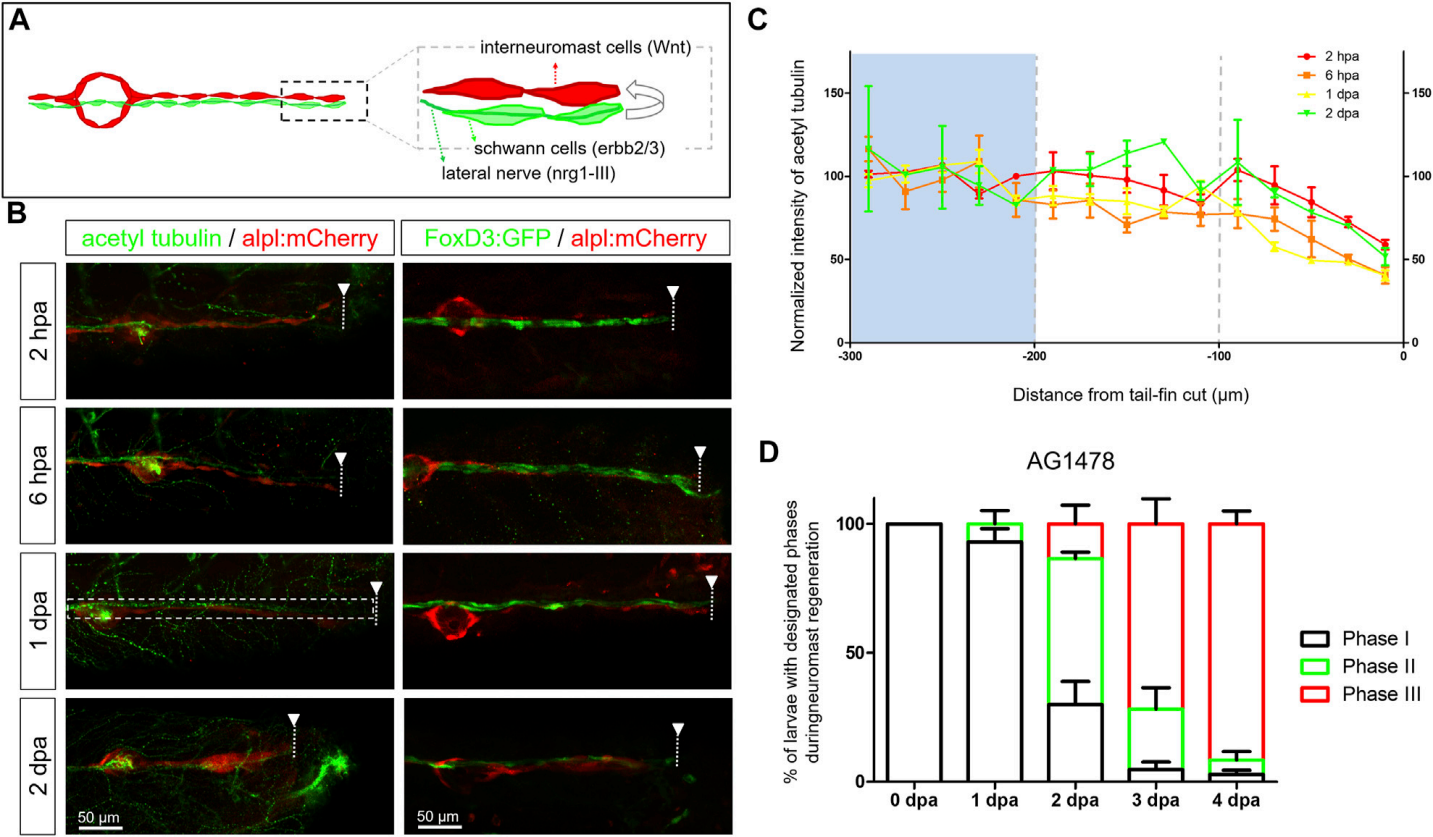
Diminished nerve inhibition near the cut site may allow neuromast regeneration from interneuromast cells. **(A)** A graph shows the close contact between interneuromast cells (INCs, red) with underlying Schwann cells (SWCs, light green) and posterior-lateral nerve (pLLn, dark green). The pLLn-derived nrg1-III can activate erbb2/3 receptors on SWCs to suppress Wnt activity in INCs, thus keeping INCs in a quiescent state. **(B)** The 3-dpf larvae from the cross of *Tg(−4.7alpl:mCherry)* and *Tg(foxd3:EGFP)* were tail fin–amputated and immobilized at the designated time to show the integrity of SWCs in green [labeled by *Tg(FoxD3:GFP)* in all stages examined (right column)]. *Tg(−4.7alpl:mCherry)* larvae at 3 dpf were also tail fin–amputated, fixed at designated hours or days post-amputation (hpa or dpa, respectively), and subjected to immunostaining against acetyl tubulin to reveal green nerves along the red lateral line (left column). The fluorescent intensity of green pLLn is diminishing, approaching the cut site (dashed line). So, we measured the green fluorescent intensity at decreasing distance from the cut site in cropped images as depicted by a dotted rectangle as shown in the image of a 1-dpa larva. The averaged intensity was calculated and normalized with those of the proximal region (200–300 μm from cutting edge, blue background) for designated times shown by different colors. **(D)** The *Et(HG7L)* larvae at 3 dpf were treated with 3 μM of AG1478 and fin-amputated to examine neuromast regeneration according to Figure 1C. Data are presented as mean ± s.e.m.

To understand how INCs become active from a quiescent state (Figure 3A), we first investigated whether pLLn or SWC was affected after tail amputation due to the reported inhibition of Wnt signaling by the epidermal growth factor receptor (EGFR) pathway between SWC and pLLn (Lush and Piotrowski, 2014). We collected larvae from the cross of *Tg (−4.7alpl:mCherry)* and *Tg (FoxD3:GFP)* (Gilmour et al., 2002) to reveal the MCs/INCs and SWCs, respectively. Larvae were fixed two hours post-amputation (hpa), 6 hpa, 1 dpa, and 2 dpa, then subjected to immunohistochemistry against acetyl tubulin to reveal the pLLn. Although we expected to see a possible retraction of injured nerves (Graciarena et al., 2014), unexpectedly, both pLLn and SWCs appeared to stay in parallel to the distal end of the lateral line (Figure 3B, dashed lines with arrowheads indicated). The SWCs showed constant fluorescent intensity. However, the pLLn exhibited a lower fluorescent signal (at least 60% compared to the proximal end), approaching the distal end (Figure 3B, left panels) by quantifying averaged intensity at the GFP channel (Figure 3B, dashed brackets) every 20 μm from the cutting edge. The reduction of signals from 6-hpa (orange curve) and 1-dpa larvae (yellow curve) showed a steeper slope (Figure 3C) compared to those at 2 hpa or 2 dpa (Figure 3C, red and green curves, respectively). These results suggest the diminishing of nerves and associated signals.

The nerve-derived EGFR signal inhibits the differentiation of INCs. We then blocked the EGFR pathway with an EGFR tyrosine kinase inhibitor, AG1478, post-fin amputation (Osherov and Levitzki, 1994). The AG1487-treated larvae appeared to have faster recovery of new neuromasts (Figure 3D). Compared to fish not exposed to AG1487, as shown in Figure 1D, the % of larvae with a new neuromast (Phase III) had a 4.7-fold (13.5% vs. 2.9%), 2.1-fold (71.8% vs. 34.2%), and 2.1-fold (91.6% vs. 44.2%) increase in AG1487-treated larvae at 2, 3, and 4 dpa, respectively (Figure 3D). Most larvae (*n* = 48) accomplished neuromast regeneration at 4 dpa. To have a more detailed observation in real-time, we monitored the neuromast regeneration process under LSFM. We observed vigorous cell divisions (Supplementary Video S4, cells before mitosis labeled by white asterisk, daughter cells marked by magenta asterisks). Besides, many cell protrusions in various directions were observed (Supplementary Video S4, arrowheads). It suggests that pLLn and SWC play a dominant role in modulating the progenitor status of INCs during development (Lyons et al., 2005; Lush and Piotrowski, 2014) and regeneration.

**FIGURE 4:**
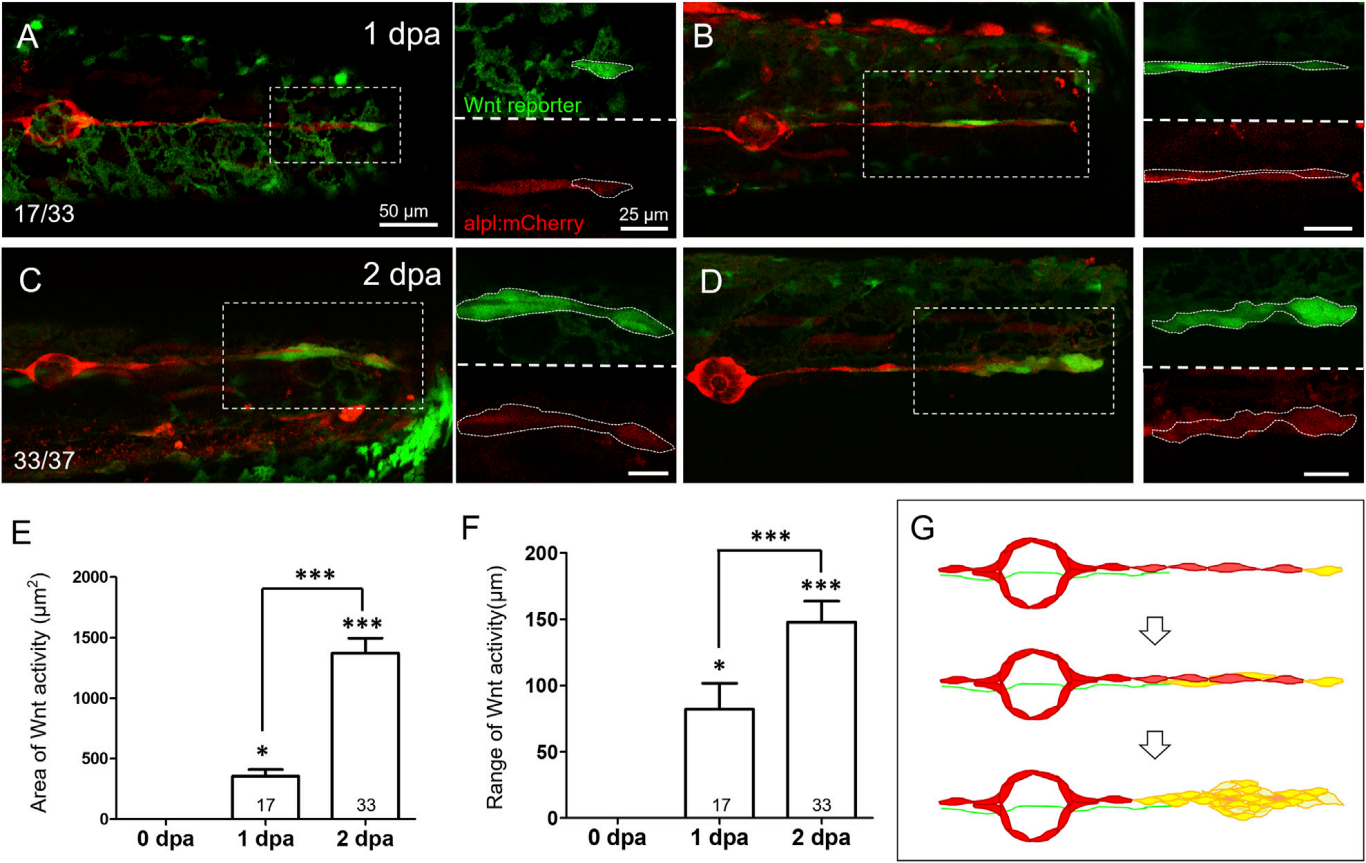
Wnt activity is enhanced in the lateral line near the injured site at 1–2 days post-injury. Representative superimposed stacked images of two different larvae **(A**‐**C)** and **(B**‐**D)** from the cross of *Tg(6XTcf/LefBS-miniP:d2GFP)* (green) and *Tg(−4.7alpl:mCherry)* (red) at 1-day post-amputation [dpa, **(A**‐**B)**] and 2 dpa **(C**‐**D)** are shown. Magnified diagrams for each dashed rectangle are shown next to the corresponding superimposed photo at the green or red channel. Elevated green Wnt activity outlined by white dashed lines was observed in the surrounding lateral line interneuromast cells near the wound site from 1 dpa. Scales are the same for all superimposed images **(A)**. Scale bars are 25 μm in magnified images. **(E**‐**F)** The areas and range of Wnt activity, defined by measuring the extension from tail-cut to the most proximal end, were calculated and shown. Data represent mean ± s.e.m. and are analyzed by one-way ANOVA compared to 0 dpa. In addition, the difference between 1 and 2 dpa was analyzed and shown. *p < 0.05, ***p < 0.0005. **(G)** A series of cartoons illustrates Wnt activity elevation (yellow) during cluster formation.

Since SWCs maintained INCs in a quiescent state by inhibiting Wnt/β-catenin signaling, we next asked whether Wnt reactivation occurs within the pLL system during regeneration. We used a Wnt reporter transgenic fish, *Tg(6XTcf/Lef-miniP:d2GFP)*, which uses a Wnt-responsive *6XTcf/Lef-miniP* promoter, to drive the expression of a degradable form of GFP, d2GFP. The fast decade of d2GFP prevents the interference of persistent GFP fluorescence and allows dynamic monitoring of Wnt activity (Shimizu et al., 2012). From 1 dpa, we noticed the elevation of Wnt activity in a subpopulation of INCs at the distal end in the tail fin-amputated larvae from the cross of the *Tg(6XTcf/Lef-miniP:d2GFP)* and *Tg (−4.7alpl:mCherry)* lines (Figures 4A and B, *n* = 17/33). From a close-up view (dashed rectangles with enlarged and split channels shown on the right-hand side), these subpopulations seemed to correspond with emerging cells that appeared besides existing ones, climbed up, and moved toward the injury site (Supplementary Video S1, arrows). Moreover, while the cluster appeared at 2 dpa, the regime of Wnt activity expanded as expected (Figures 4C and D, *n* = 33/37), with a significant increase in area and range (defined as the distance between the proximal and distal end of d2GFP signals) (Figures 4E and F). These observations suggest the elevation of Wnt activity within INCs post-fin amputation (Figure 4G).

### Activation of Interneuromast Cells Even in the Presence of Intact Schwann Cells and Lateral Nerves

**FIGURE 5:**
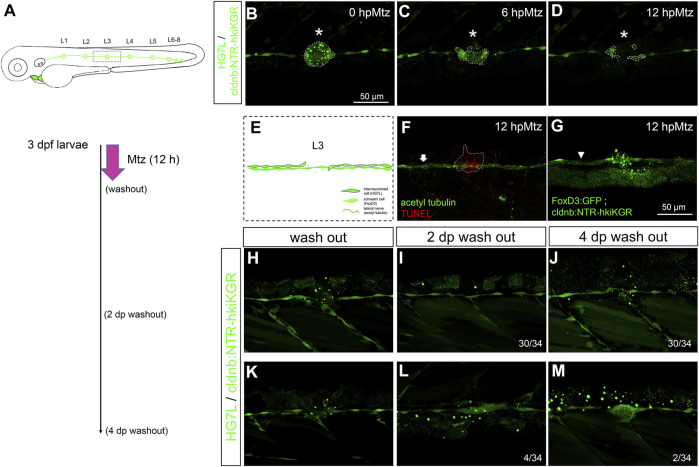
Chemical-genetic ablation of neuromasts blocks neuromast regeneration in most but not all larvae. **(A)** Larvae from the cross of *Et(HG7L)* (green) and *Tg(−8.0cldnb:NTR-hkiKGR)* (green dots) were treated with 2 mM metronidazole (Mtz) ob 3 days post-fertilization (dpf) for 12 h, washed out, cultured, examined, and photographed at designated h post-Mtz treatment (hpMtz) as shown in a series of cartoons. **(B–D)** Representative stacked images show decreasing size (enclosed by dashed lines) of neuromast (asterisks) during Mtz treatment. **(E)** A cartoon shows the absence of neuromast with undamaged Schwann cells and lateral nerve. **(F)** The TUNEL staining clearly labeled apoptotic cells in the Mtz-treated larva, and the lateral nerve (arrow) stayed undamaged. **(G)** Mtz was used to treat larvae from the cross of *Tg (foxD3: GFP)* and *Tg (−8.0cldnb: NTR-hkiKGR)*. In a representative stacked image, Schwann cells (arrowhead pointed green rod) appeared intact compared to the disintegrating neuromast (asterisk). The faint broad green signal underneath is stacked signals from underlying tissues. **(H–J)** After washing out Mtz, most of the interneuromast cells (30/34) in the proximity of ablated neuromasts did not form a new neuromast. **(K–M)** A few (2/34) larvae had regenerated a neuromast **(M)** through cluster formation (4/34) **(L)**. Scale bars are the same in all panels but are only shown in **(B)**.

Copper sulfate at 100 μM could destroy a whole neuromast without harming the nearby nervous system (Hernández et al., 2006; Hernandez et al., 2011). The neuromast cannot regenerate under this “more” specific ablation (Sánchez et al., 2016). It supports the overwhelming inhibitory signals from pLLn and SWC on the regeneration capacity of INCs. We used the Tg(*−8.0cldnb:NTR-hKikGR*) line to test further this hypothesis, which explicitly expresses NTR in the lateral line (Supplementary Figure S3). More importantly, we identified a founder fish carrying stronger NTR-hKikGR signals in neuromasts than in INCs (Supplementary Figure S3B). This line allowed us to kill neuromasts without damaging INCs chemically. To validate this chemical ablation method, we crossed this fish with *Et(HG7L)* to reveal the pLL system and then treated the larvae with metronidazole (Mtz) at 3 dpf (Figure 5A). The green fluorescence in neuromast cells gradually abolished, while INCs remained unchanged within a half-day incubation of Mtz (Figures 5B–E). The loss of fluorescence implied the death of cells. By TUNEL assay, we found that cell apoptosis only occurred within neuromasts but not INCs, SWCs, and the pLLn in Mtz-treated larvae (Figures 5F–G). Therefore, this NTR/Mtz ablation system appears to be a practical approach to killing cells in neuromasts without affecting nearby cells specifically.

We thus incubated 3-dpf larvae from the cross of *Tg(−8.0cldnb:NTR-hKikGR)* and *Et(HG7L)* lines with Mtz for 12 hours and then washed them out to see whether the ablated L3 neuromast could be regenerated. We observed the healing of the injured lateral line, but most fish (30/34) did not restore the L3 neuromast even 4 days after the washout of Mtz (Figures 5H–J). However, a few larvae (4/34) (Figure 5K) still formed a cluster two days post-Mtz washout (Figure 5L), and two of them (2/34) even regenerated neuromasts four days post-washout (Figure 5M). It suggests other factors might override the inhibition from pLLn/SWC to activate INCs.

### Macrophages Relieve the Neural Inhibition of Interneuromast Cells

Leukocyte infiltration is the first line of the innate immune response. We hypothesized that leukocyte infiltration might involve in the regulation of neuromast regeneration. Using *Tg(mpx:EGFP)* larvae, we observed that neutrophil recruitment reaches a peak at 6 hpa and declines within 1 dpa. The recruitment of neutrophils was significantly reduced in larvae treated with diclofenac (3 μM), known to inhibit neutrophil recruitment (Carrillo et al., 2016; d’Alençon et al., 2010), as shown in Supplementary Figure S4A. We also observed a reduction of neutrophil recruitment at 6 h post-Mtz in amputated larvae (Supplementary Figure S4B). We further inhibited neutrophil recruitment by treating tail-amputated 3-dpf larvae with diclofenac but still observed a similar regime of neuromast regeneration (Supplementary Figure S5A). We used clodronate-loaded liposomes (clodrosomes) to test macrophage involvement, which induces apoptosis of phagocytes (van Rooijen and Hendrikx, 2010; Carrillo et al., 2016). Clodrosomes effectively eliminated the resident macrophages in larvae from a *Tg (mpeg1: mCherry)*, a macrophage reporter line (Ellett et al., 2011) (data not shown). We thus injected control liposomes, or clodrosomes, into blood circulation *via* the heart of a 3-dpf larva before tail amputation. Interestingly, the regeneration process was slowed down, with less than half of larvae (11.5%) regenerating a new neuromast at four dpa in clodrosome-treated larvae (Supplementary Figure S5B), supporting the involvement of macrophages in neuromast regeneration upon tail amputation.

**FIGURE 6:**
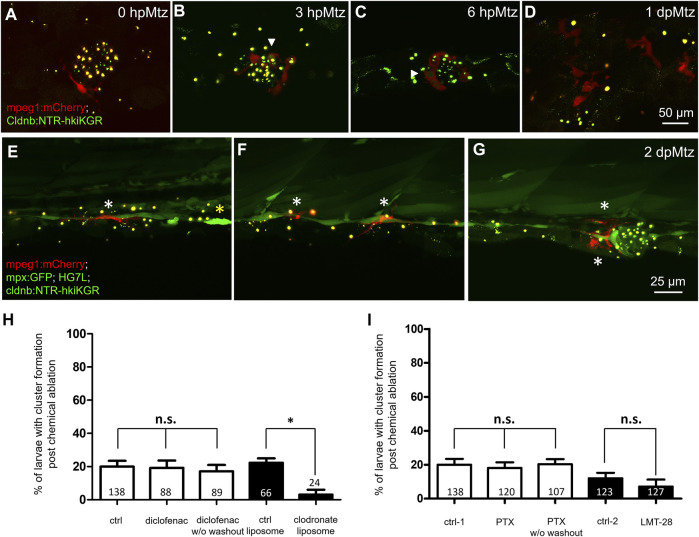
Changes in phagocytes surrounding deteriorating neuromasts and their effects on cluster formation. Larvae from the cross of *Tg (mpeg1: mCherry)* (red) and *Tg(−8.0cldnb:NTR-hkiKGR;myl7:EGFP)* (green) were treated with metronidazole (Mtz) as described in Figure 5, examined and photographed at designated hours or days post-Mtz treatment (hpMtz or dpMtz). We observed the disintegration of neuromasts post-Mtz treatment **(A–D)**. Red macrophages (arrowheads) were recruited to engulf injured neuromasts in a dendritic cell-like shape within hours post-Mtz treatment **(A–C)**. The injured neuromast disappeared, but macrophages were still retained in the injured site at 1dpMtz. Please note that the macrophages became more compact round in shape with protrusions **(D)**. **(E–G)** Triple-transgenic larvae as designated were treated as described above, examined and photographed at 2 dpMz. Macrophages (red, white asterisks) were still found in the posterior lateral line system, even in a new neuromast, as shown in **(G)**. In contrast, only a few neutrophils (bright green, yellow asterisks) were found. Scales are the same for each row but are only shown in the far-right panel. **(H)** We used larvae from the cross of *Et(HG7L) and Tg(−8.0cldnb:NTR-hKikGR;myl7:EGFP)*. The larvae were treated without (ctrl) and with 3 μM diclofenac (with or without washout); control or clodronate liposome **(H)**; or treated with different cytokine inhibitors PTX (35 μM) or LMT-28 (10 μM) **(I)**. The above-treated larvae were undergone a 12-h Mtz treatment and scored for the % of larvae with cluster formation. Data represent mean ± s.e.m. and were analyzed by one-way ANOVA, *p < 0.05. n.s.: not significant.

To test whether macrophages are involved in the residual regeneration capacity seen in Mtz-treated larvae retaining potent inhibition from intact SWCs, we treated larvae with diclofenac or clodrosomes before Mtz treatment. We found significantly hampered neuromast regeneration only by inhibiting macrophages (Figure 6H). It suggests the role of macrophages in alleviating the inhibition signals from pLLn. Using larvae from the cross of *Tg (HG7L; −8.0cldnb: NTR-hKikGR)* and *Tg (mpeg1: mCherry)*, we observed that some resident macrophages initially positioned around neuromast (Figure 6A), then were recruited to the injured neuromast with lobulated morphology (Figures 6B–D). Polarization status is a critical feature of M1 or M2 macrophages (Nguyen-Chi et al., 2015). The M1 macrophages act as pro-inflammatory cells to engulf apoptotic cells (indicated by arrowheads as green signals within red cells). In a later phase, M2 macrophages, transformed from the M1 status, display dendritic morphology and are essential for inflammation resolution (anti-inflammatory) and tissue modeling. At 2 dpMtz, many elongated macrophages were still there to interact with the injured pLL system compared to only a few neutrophils (yellow asterisk) existed in larvae from the cross of *Tg(mpeg1:mCherry;mpx:GFP)* and *Tg(−8.0cldnb:NTR-hKikGR;HG7L)* (Figures 6E–G).

Recently, accumulating evidence points to the pivotal role of macrophages in wound repair and tissue regeneration, mainly *via* secreted cytokines (Zhang et al., 2018). We thus blocked several cytokines secreted by macrophages, such as tumor necrosis factor-alpha (TNFα) and interleukin-6 (IL-6). Pentoxifylline (PTX) is known to inhibit *tnfα* transcription (Doherty et al., 1991; Schmidt-Choudhury et al., 1996; El-Ghoneimi et al., 2007). LMT-28 can block the IL-6 receptor β subunit (glycoprotein 130, gp130) (Hong et al., 2015a). Compared to the designated DMSO or methanol control, neither PTX nor LMT-28 treatment ruined the enduring capacity for neuromast regeneration at the dosages tested (Figure 6I).

### Macrophages Patrol in Between Injury Sites More Often in the Later Regeneration Phase

**FIGURE 7:**
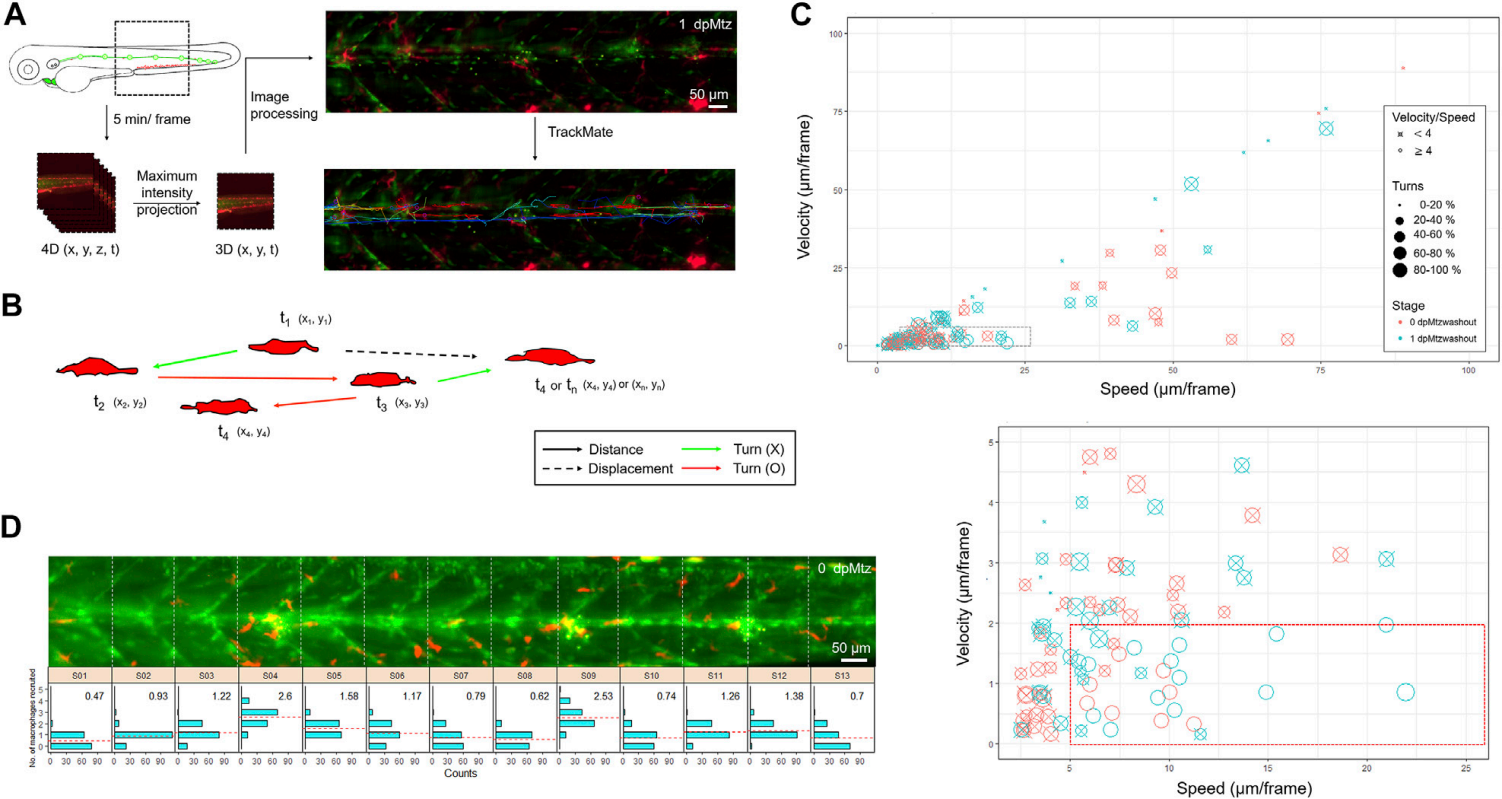
*In toto* imaging analysis reveals differential cell behaviors and specific spatial distribution of macrophages during neuromast regeneration. **(A)** The flowchart shows that the trunk region, including neuromast L3-5 was imaged *in-toto* by light-sheet fluorescence microscopy (LSFM) to monitor macrophage behavior during neuromast regeneration. Larvae from the cross of *Tg(mpeg1:mCherry;FoxD3:GFP)* and *Tg(−8.0cldnb:NTR-hKikGR); Et(HG7L)*, a wide field of z-stack images focusing around the cloaca (dashed rectangle) was taken every 5 min. We then reduced the order to 3D (x,y,t) with the stacking of z-sections and processed it to have a better resolution by subtracting the background. Finally, we used the plugin “TrackMate” in the Fiji software and manually modified it to acquire the tracking path of every macrophage (shown in the right-bottom corner). **(B)** We measured the distance (arrows) and displacement (arrows with dashed lines) to calculate speed and Velocity, as shown in **(C)**. Turns were counted among cell movements (indicated by red) to generate the ratio of turns in **(C)**. **(C)** A scatter plot shows specific speed (the X-axis) and Velocity (the Y-axis) for each macrophage. Other features such as “Speed/Velocity”, “Ratio of turns” and “Stage” are also depicted as shown in the legends on the right to distinguish different cell behaviors better. A region with a speed lower than 25 and a velocity smaller than 10 (dashed rectangle) was magnified, as shown in the lower panel. **(D)** Histograms divided by sections (width of 100 μm) represent counts (the X-axis) of the different numbers of recruited macrophages (the Y-axis) during neuromast regeneration. In each panel, the mean of the “number of recruited macrophages” is shown in the right-upper graph and presented with dashed lines in red.

To further understand the interaction between macrophages and regenerating neuromasts, we aimed to dissect their spatial and temporal relationship more thoroughly. First, the larvae from the cross of *Tg(mpeg1:mCherry;FoxD3:GFP)* and *Tg(−8.0cldnb:NTR-hKikGR;Et(HG7L))* were treated with Mtz and examined under LSFM. We monitored the larvae in a wide field of view (1.3 mm × 1.3 mm), focusing on neuromast L3 to L5, with about 200 z-sections (step size of 1 μm) every 5 min (Figure 7A). For analytic convenience, we stacked the z-sections by maximum intensity projection and then cropped the region of interest (ROI) for further image processing. We used the plug-in “TrackMate” in Fiji software (Tinevez et al., 2017) to analyze mCherry-marked macrophages under a red channel with manual corrections of spot labeling to indicate the center of a macrophage and spot linkage to show the migrating route of a macrophage at two continuing time points (Figure 7A). We also excluded macrophages that did not contact the fluorescent pLL from the HG7L line by built-in filters. We show every tracking path of an individual macrophage by its index or displacement with a color map (Figure 7A, right). By recording the location of each macrophage at different time points (X_n_, Y_n_, T_n_), we acquired valuable features to distinguish specific behaviors (Figure 7B). We calculated “displacement against distance” by the difference between the final and initial positions or the sum of all movements. We also provide the velocity or speed of individual macrophages.

In Supplementary Video S5, we show a representative *in toto imaging movie*. The video stacks images from different sections. We merged images acquired from the red (showing macrophages) and green channels (showing lateral line cells and SWCs). To facilitate the catching of macrophages, we label macrophages with purple circles and mark their migrating traces. Macrophages migrate toward and around injured neuromasts in different dynamics during regeneration. Therefore, we analyzed movies from two larvae, each at 0 and 10 h post-Mtz washout. As expected, some macrophages resided at the injury site for a long while (Supplementary Figure S6, arrows). In contrast, other macrophages patrolled across the injured area at different ranges (Supplementary Figure S6, arrowheads), and a few of them passed by rapidly (Supplementary Figure S6, asterisks). The “patrol” behavior is characteristic of a high speed–velocity ratio and frequent direction turns (Figure 7B, red arrows). We used a scatter plot to represent different cell behaviors of all macrophages in the whole-trunk region of Mtz-treated larvae (Figure 7C). Each circle represents one macrophage. More than half of the macrophages moved gently and slowly (0.2∼2 μm/min). In contrast, a population of actively moving macrophages (speed: > 1 μm/min, velocity: < 0.4 μm/min, speed/velocity > 4, ratio of turns > 40%) increased significantly in the later regenerative phase at 1-day post-Mtz washout (Figure 7C, marked by a red rectangle in dashed line at the bottom). Other macrophages outside of this group are marked out with an “X” for better visualization. This increase in active macrophage migration might be relevant to neuromast regeneration.

To quantify the distribution of macrophages and the pLL system further, we videotaped an Mtz-treated larva at 5 minutes per frame from 0 to 12 h post-Mtz washout (Supplementary Video S6). For analysis, we divided the trunk into 13 sections (S01–S13, each with 100 μm in width) according to the chevron-shaped muscle segment marked in the *Et(HG7L)* line. We counted the number of recruited macrophages in each section at each frame and presented a histogram to show the distribution and average numbers of macrophages accumulated from all frames (Figure 7D). We found more accumulated macrophages in sections containing injury sites (S04, S09, S11, and S12) but uneven distribution of macrophages were found in those uninhured sections (S5: 1.58 and S6: 1.11 vs. S7: 0.79 and S8: 0.62). It indicates the injury-induced recruitment of macrophages and implies a possible involvement of macrophages in neuromast regeneration. Altogether, we hypothesize that the more frequent macrophage patrolling around injured neuromasts in the later regeneration phases may account for the residual regeneration capacity in Mtz-treated larvae.

### Macrophage Intervention may Relieve the Neural Inhibition of Interneuromast Cells

**FIGURE 8:**
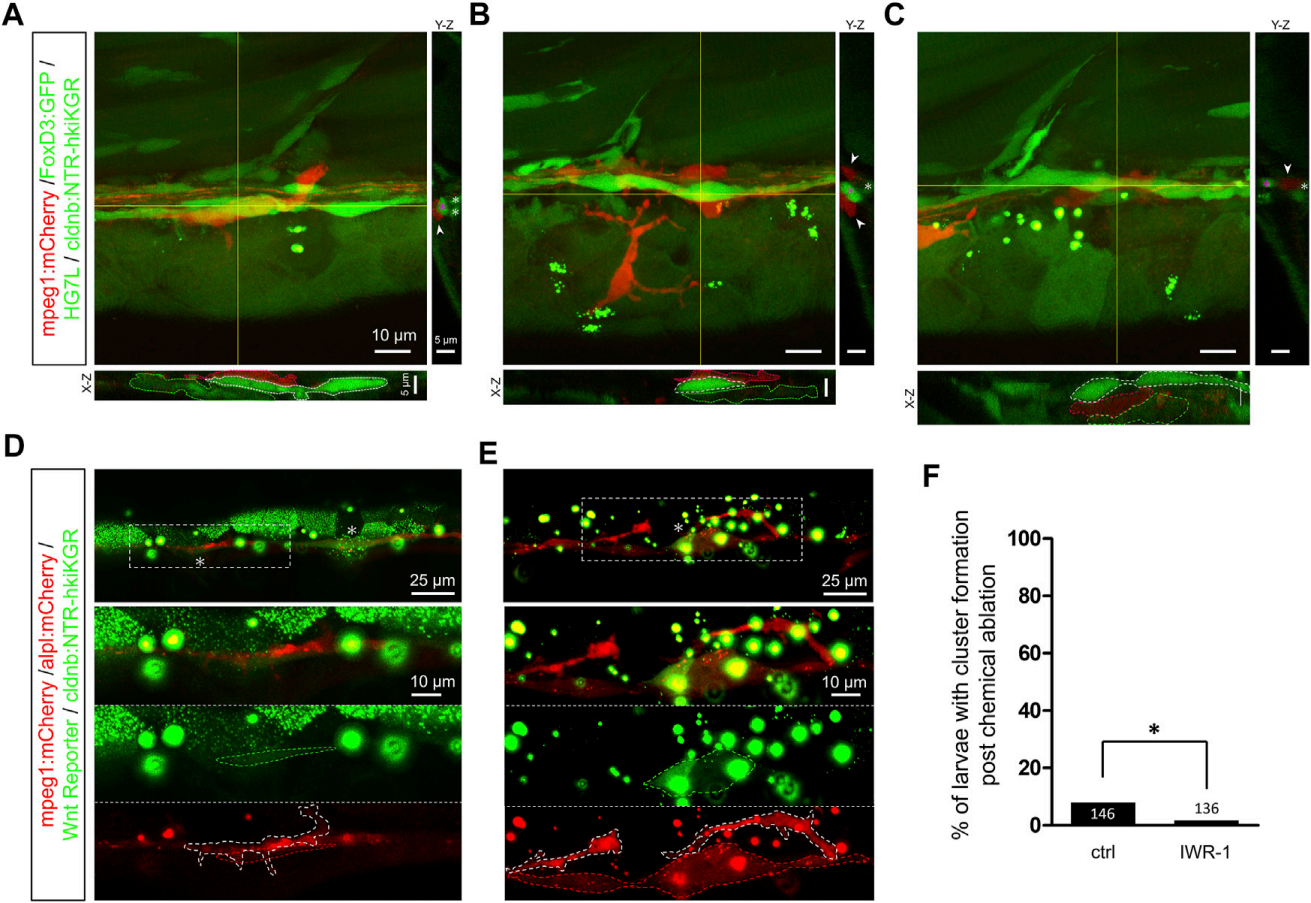
Infiltrated macrophages are found between Schwann cells and interneuromast cells with higher Wnt activity in Mtz-treated larvae. **(A–C)** Images shown are representative confocal stacked images of Mtz-treated larvae from the cross of *Tg(mpeg1:mCherry;FoxD3:GFP)* and *Tg[−8.0cldnb:NTR-hKikGR;Et(HG7L)]* in orthogonal views, while the X-Z and Y-Z views at the position of yellow lines are shown below and on the right side, respectively. Macrophages (red) indicated by red dashed outlines were mostly crawling on interneuromast cells (INCs, brighter green, marked by white dashed outlines) in **(A–B)**. In some cases, macrophages were found between INCs and Schwann cells (SWCs, dim green, labeled by green dashed outlines) **(C)**. Scale bars are the same but only provided in **(A)** for respective images. **(D** and **E)** Images presented are representative stacked confocal images of Mtz-treated larvae from the cross of *Tg (mpeg1:mCherry;−8.0cldnb:NTR-hKikGR)* and *Tg(−4.7alpl:mCherry;6XTcf/LefBS-miniP:d2GFP)*. Areas shown higher Wnt activity are boxed with dash lines and indicated by asterisks. Magnified diagrams for the dashed rectangles are shown in different color channels to reveal INCs (red, top panel), Wnt signal (dim green, dashed region, middle panel), and macrophage (red, dashed region, bottom panel). Interestingly, some macrophages (enclosed with a white dashed line) interacted with these activated INCs. The scale bar goes to 25 μm or 10 μm in magnified diagrams. **(F)** Larvae were treated without (Ctrl) or with IWR-1 to disrupt Wnt activity, and the percentages of larvae with cluster formation were counted as described previously. Data represent mean ± s.e.m. and were analyzed by one-way ANOVA, *p < 0.05.

By closely examining the *in toto* time-lapse movies through the Z-axis, we found macrophages from the top surface to the bottom of the trunk (Supplementary Video S7). Macrophages often crawled on INCs with nearby SWCs. Interestingly, some macrophages protruded into the limited space between INCs and SWCs. To go more in detail, we thus examined this hierarchical structure under confocal microscopy at higher magnification using a 63X oil lens to dissect the positions of macrophages, INCs, and SWCs along the Z-axis and further clarification in orthogonal views (Figures 8A–C). Commonly, macrophages lay on top of INCs and SWCs, as shown in Figures 8A and B. However, macrophages could squeeze into the tiny space between INCs and SWCs, as demonstrated in Figure 8C. It implies that the macrophage may interfere with the physical contact between INC and SWC.

Upon the second lateral line development, the first lateral line’s INCs are pushed ventrally by the migrating 2^nd^ primordium to be far away from the inhibitory signal of SWCs. It activates the quiescent INCs to form intercalary neuromasts. It suggests the close relationship between INCs and SWCs is critical to keeping INCs silent. We thus hypothesized that the macrophage infiltration could step in to block the neural inhibition. To test this, we first analyzed whether Wnt activity is upregulated in the Mtz-treated larvae. Indeed, we saw increased Wnt activity in lateral line cells or cell clusters during regeneration in Mtz-treated larvae from the cross of *Tg(mpeg1:mCherry;−8.0cldnb:NTRhKikGR)* and *Tg(−4.7alpl:mCherry;6XTcf/LefBS-miniP:d2GFP)* (Figures 8D and E). Interestingly, some macrophages (enclosed in white dashed line) were in close physical contact with INCs (encircled in red dashed line) with upregulated Wnt activity (circled in green dashed line) (Figures 8D and E, lower panels). Furthermore, the inhibition of Wnt signaling by IWR-1, a β-catenin complex stabilizer (Chen et al., 2009), almost completely abolished the residual regeneration capacity (Figure 8F). It implies the effect of macrophages works through the activation of INCs with Wnt signaling.

## Discussion

The keys to unraveling the mystery of regeneration are identifying progenitor cells complementing the loss of organs and the underlying induction mechanism therein. This study removed the L6-8 lateral line neuromast in zebrafish larvae by tail amputation or genetic-chemical ablation of the L3 neuromast. We found the nearby INCs unequivocally can be activated to form a new neuromast. The activation of INCs is at least partly by alleviating the inhibition from pLLn/SWC *via* the intervening infiltration of macrophages.

We observed three sequential phases constituting the whole regeneration morphogenesis of neuromast, as reported previously (Moya-Díaz et al., 2014; Sánchez et al., 2016). Our data agree with Sanchez et al. (2016) that INCs are multipotent progenitors for neuromasts by indirect labeling or cell transplantation. Furthermore, EGFR signaling is the factor for keeping the quiescent status of INCs since they can regenerate by inhibiting EGFR signaling with AG1478 (Sánchez et al., 2016). However, we observed no gap-filling activity before 1-day post-injury, in contrast to the work by Sánchez et al. (2016). Electroablation might cause an instant injury of the underlying SWCs and pLLn. As in our case, the diminishing signal of pLLn in the nearby region of a cutting site might indicate a lower expression of Neuregulin 1 type III (Nrg1-3), which is involved in the migration, proliferation, and differentiation of SWCs (Perlin et al., 2011) *via* the receptor, ErbB2 or ErbB3, of SWCs (Lyons et al., 2005; Rojas-Muñoz et al., 2009). This tripartite relationship is well-established to explain precocious intercalary neuromast formation with the interruption of this ErbB/Neuregulin pathway (Lush and Piotrowski, 2014). Whichever mechanisms they adopted, INCs could respond to neuromast ablation as multipotent progenitors by proliferating and replenishing the loss of an organ.

Tissue damage, either sterile or infectious, usually accompanies innate immunity to protect the organism at the first front line. This inflammatory response features sequential recruitment of different leukocytes to the injury site (Keightley et al., 2014); neutrophils are pioneers, while macrophages arrive later as a typical immune process seen previously in zebrafish (d’Alençon et al., 2010; Loynes et al., 2010; Li et al., 2012) and also evident in our study. Neutrophils can eliminate pathogens by phagocytosis and maintain inflammation with IL1-β secretion (Kolaczkowska and Kubes, 2013). This inflammation is augmented by myeloid cells and surrounding injured cells. They further recruit macrophages to participate in a positive feedback loop.

Moreover, our data reconfirm macrophage morphology changes from round to dendritic shape during regeneration. The M1-like (pro-inflammatory) macrophages almost switch their polarity to M2-like (non-inflammatory) (Sica and Mantovani, 2012) within 1-day post-injury. This transition, also known as inflammation resolution, was triggered by the inhibition of IL1-β signaling through TNFα secretion (Tsarouchas et al., 2018) and efferocytosis, a process by which activated or infected neutrophils are engulfed by M1-like macrophages (Martin et al., 2014). Thus, prolonged inflammation is detrimental since it induces more apoptosis and threatens regeneration (Hasegawa et al., 2017). Interestingly, M1-like macrophages could promote zebrafish fin regeneration through TNFα/TNFR1 on blastema cell proliferation (Nguyen-Chi et al., 2017) and stimulate myogenic precursor cells in mammals to divide through secretion of TNFα, IL1-β, and IL-6 (Arnold et al., 2007; Saclier et al., 2013a; Saclier et al., 2013b). This promotion likely relies on alleviation of IL1-β signaling, achieving inflammation resolution, instead of cell debris clearance or cell death prevention (Tsarouchas et al., 2018). On the other hand, being anti-inflammatory, M2-like polarized macrophages could also be involved in inflammation resolution *via* secretion of anti-inflammatory molecules, such as TGF-β1 or IL-10, and further tissue remodeling (Nguyen-Chi et al., 2015; Nguyen-Chi et al., 2017). Nguyen-Chi et al. (2017) treated amputated-larvae from *Tg(mpeg1:mCherry-F/tnfa:eGFP-F)* with 35 µM pentoxifylline (PTX). They found that the PTX treatment decreased GFP-F+ cells (TNFa expressing cells, especially GFP-F+ macrophages) in the fin at 6 hpa. The number of recruited macrophages at the wound was lower at 24 hpa. Furthermore, they observed the decrease of *tnfʱ* mRNA expression in PTX-treated fins at 5 *hpa*. Although they also discovered the reduction of *il1b* but to a lesser extent. We adopted the reported PTX concentration (35 µM) used and found that the residual regeneration capacity was not affected by inhibiting TNFα signaling. It suggests that M1-like macrophages may not play an essential role through the secretion of TNFα. However, the effects of PTX on the TNFα signaling like the downregulation of *tnfα* mRNA expression should be demonstrated for definite proof. Similarly, although we observed no effect on the enduring capacity for neuromast regeneration by LMT-28 at a dosage effectively blocking IL-6 activity in cellular studies (Hong et al., 2015b) that suggests the macrophage may modulate neuromast regeneration independent of IL-6. Proof of LMT-28 IL-6 blocking activity in zebrafish embryos is still lacking. Moreover, studies of the effects of more cytokines and combinatory cytokines are also needed to define the involvement of cytokines. Furthermore, we need investigations like the ablation of macrophages at different time points after injury or transgenic line, *Tg (tnfa: EGFP-F),* to distinguish two classes of macrophages (Nguyen-Chi et al., 2015) to resolve the contribution of M1-like or M2-like macrophages in the neuromast regeneration.

We performed macrophage tracking post-injury with wide-field acquisition and categorized individual cell behaviors as “Stay”, “Patrol”, and “Flash”. Previous studies provided velocity and speed data to record immune cell movement during fin or retina regeneration (Nguyen-Chi et al., 2015; d’Alençon et al., 2010; Li et al., 2012; White et al., 2017; Morales and Allende, 2019). They found cell heterogeneity between peripheral and resident macrophages (Morales and Allende, 2019) or macrophage’’ behaviors upon different types of injuries (White et al., 2017). We introduced more parameters revealing direction (velocity/speed), orientation (turns), and phase to identify novel cell subclasses. While patrolling macrophages increase in the later regeneration phase (1-dpMtz), all three behaviors appeared in the early (0 dpMtz) and late stages. This novel category may not correspond precisely to the M2-like macrophages, which could be subdivided into M2a, M2b, and M2c subclasses in mammals (Rőszer, 2015).

Furthermore, macrophages seem to stay in hot zones and shuttle between SWCs and INCs with high z-resolution analysis. Thus, we hypothesize that more certain M2-like macrophages (1-dpMtz) could interrupt the connection between SWCs and INCs in the specific region *via* an unknown mechanism. The dissociation ability could be either physical or chemical. Macrophages could generate physical force to adhere to and connect the ruptured blood vessels (Liu et al., 2016). We also witnessed a macrophage pushing away INCs from underlying SWC and nerve while transpassing.

Further *in-vivo* dynamics of actin filaments regulated by phosphatidylinositol-3-kinase (PI3K) or Ras-related C3 botulinum toxin substrate 1 (Rac1) could help reveal the filopodia or lamellipodia-dependent cellular action. A chemical reaction could be extracellular matrix (ECM) remodeling by matrix metalloproteinases (MMPs) from macrophages (Jenkins et al., 2016). ECM remodeling further mediating leukocyte recruitment was regulated by critical expression of MMPs family enzymes such as MMP-9 or MMP-13 and was crucial to heart regeneration in zebrafish (Xu et al., 2018). Our preliminary data showed that macrophages interact with the 2nd pLLp migration (Supplementary Figure S7; Supplementary Video S8). Interestingly, since macrophages are supposedly gifted to separate INCs and SWCs, they were found to visit along the migratory trajectory of 2^nd^ pLLp frequently. Whether macrophages promote intercalary neuromast formation *via* dissociation of SWCs and 1^st^ lateral line needs to be further investigated. Therefore, we hypothesize that macrophages could play an essential role in separating INCs and SWCs both in development and regeneration. While macrophages could digest the linkage between SWCs and the 1^st^ lateral line, the 2^nd^ primordium could help create enough space to alleviate the inhibition from SWCs, resulting in robust intercalary neuromast formation. During regeneration, M2-like macrophages could only separate SWCs and INCs by themselves. Limited freedom of neural inhibition with minimal gap between INCs and SWCs generated by individual macrophages thus leads to a low success rate of regeneration. Macrophages are recently well-known to participate widely in development, homeostasis, and regeneration (Stefater et al., 2011; Wynn et al., 2013). We suggest that macrophages could support different scenarios, that is, regeneration and development in zebrafish lateral line, by utilizing a similar mechanism.

**FIGURE 9:**
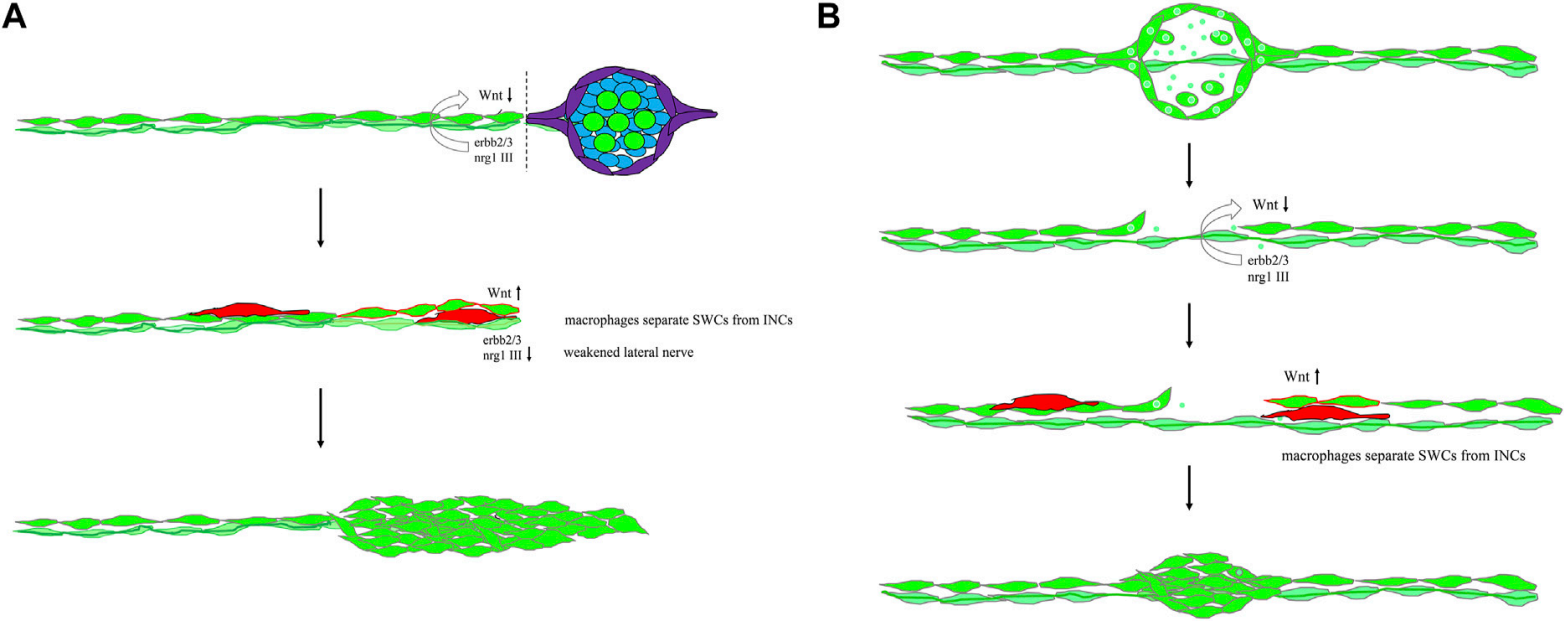
Mechanistic models for lateral line regeneration. **(A)** Under normal conditions, the pLLn (thick dark green thread) nrg1-III-activated erbb2/3 receptors within Schwann cells (SWCs, light green) keep interneuromast cells (INCs, bright green) quiescent by limiting the Wnt activity. Upon the loss of distal neuromasts by tail amputation, the lateral line nerve is weakened (thin light green thread) in the proximity of the cutting edge to break the quiescence of INCs (red outlines). The Wnt activity in INCs is elevated and results in cluster formation. **(B)** Upon specific ablation of neuromasts by NTR-hKikGR protein (bright green spots)-induced by Mtz without damaging surrounding posterior lateral line nerve and SWCs, INCs remain quiescent by integral EGF inhibition. Most macrophages crawl onto INCs, filling the gap without successful neuromast regeneration. However, occasionally macrophages could infiltrate in between INCs and SWCs. It breaks the contact between INCs and SWCs and the inhibition of EGF signaling. INCs are then activated to be able to differentiate and regenerate new neuromasts through cluster formation.

In conclusion, the inhibition from SWCs and lateral line nerves is the key factor keeping the quiescence of INCs. The quiescence of INCs can be alleviated by fin amputation or possibly *via* intervening macrophage in between the lateral line and SWCs (Figure 9). In both cases, neuromast regeneration can be blocked or delayed by inhibiting macrophages. Macrophages may also be involved in intercalary neuromast formation during development. Altogether, our results strongly suggest that macrophages may participate in neuromast development. More importantly, they play a pivotal role in awakening interneuromast cells to regenerate neuromasts in an injured lateral line in zebrafish.

## Materials and Methods

### Zebrafish Strains

Wild type AB zebrafish (*Danio rerio*), *Tg (−4.7alpl:mCherry)* (Steiner et al., 2014), *Tg(−8.0cldnb:lyn-GFP)* (Haas and Gilmour, 2006), *Tg(FoxD3:GFP)* (Gilmour et al., 2002), *Tg(mpeg1:mCherry)* (Ellett et al., 2011), *Tg(mpx:GFP)* (Renshaw et al., 2006), and *Tg(6XTcf/LefBS-miniP:d2GFP)* ^*isio1*^ (Shimizu et al., 2012) fish were maintained at 28.5°C on a 14-h light/10-h dark cycle. We constructed a transgenic cassette by combining p5E-*hsp70*, pME-EGFP, and p3E-polyA into pDestTol2CG2 *via* Gateway recombination cloning (Kwan et al., 2007). This hsp70-EGFP vector (HG) is suitable for an enhancer trap screen (Supplementary Figure S1A) (Nagayoshi et al., 2008). We conducted an enhancer trap line screen and identified an *Et(HG7L)* trap line. It was named after its specific expression in the pLL system (Supplementary Figures S1G–M). To generate the *Tg(−8.0cldnb:NTR-hKikGR;myl7: EGFP)* line, we constructed a cassette for transgenesis by combining p5E-*8.0cldnb* (a kind gift from Dr. Tatjana Piotrowski) (Romero-Carvajal et al., 2015), pME-NTR-hKikGR (a kind gift from Dr. Chung-Der Hsiao) (Chen et al., 2011), and p3E-polyA into pDestTol2CG2 through Gateway cloning. For transgenesis, Tol2 transposon and Tol2 transposase mRNA were injected at 25 pg into 1–2 cell stage embryos and raised to adults (F0). We backcrossed F0 founder fish with wild-type fish, screened F1 embryos with strong EGFP signals in the heart, and selected one founder with a strong expression in the pLL system, as shown in Supplementary Figure S3. We also generated double transgenic lines, including *Tg(−8.0cldnb:NTR-hKikGR);Et(HG7L)*, *Tg(mpeg1:mCherry;FoxD3:GFP)*, *Tg(mpeg1:mCherry;−8.0cldnb:NTR-hKikGR)*, *Tg(−4.7alpl:mCherry;6XTcf/LefBS-miniP:d2GFP), and Tg(mpeg1:mCherry;mpx:GFP)*, to be used for producing quadruple transgenic larvae as indicated. Embryos collected from natural mating were cultured and staged according to Kimmel et al. (1995).

### Whole-Mount *In Situ* Hybridization

We cloned fragments of *sorcs3* and *ccdc147* from zebrafish cDNAs by RT-PCR and subcloned them into pGEMT-easy vectors for antisense probe synthesis. Primer pairs used are as follows: *sorcs3* (forward: GTC​GCC​AAT​GCA​AGT​GAA​TTA​CGC; reverse: TTT​CCA​GAC​CAG​TAC​ACG​ACT​GCG​T) and *ccdc147* (forward: GAC​GAC​AGT​ACG​TTG​GAA​ACC​ATG​G; reverse: CGG​TGG​CTT​TAG​TAA​GGT​TTT​CCC​G). Whole-mount *in-situ* hybridization (WISH) was performed as described using digoxigenin (DIG)-labeled antisense RNA probe (Thisse and Thisse, 2008). Stained embryos were mounted in glycerol, observed under a Leica S8AP0 stereomicroscope (Leica Microsystems, Wetzlar, Germany), and photographed using a Canon 7D DSLR camera (Canon, Tokyo, Japan).

### Immunohistochemistry and Confocal Microscopy

Whole-mount immunohistochemistry (IHC) staining was performed as previously described (Inoue and Wittbrodt, 2011) by using either mouse anti-GFP antibody (GT859, GeneTex, 1:500), rabbit anti-histone H3S10ph (phosphor Ser10) antibody (GTX128116, GeneTex, 1:1000), mouse anti-ZO-1/TJP1 antibody (33-9100, Thermo Scientific, 1:100), rabbit anti-GFP antibody (GTX113617, GeneTex, 1:500), mouse anti-tubulin (acetyl Lys40) antibody (32-2700, Thermo Scientific, 1:1000), and rat anti-mCherry antibody (M11217, Molecular probes, Thermo Scientific, 1:300). Secondary antibodies used are goat anti-mouse or anti-rabbit IgG conjugated with Alexa Flour 488 or Alexa Fluor 568 (Molecular probes, 1:500). Confocal images were collected utilizing LSM 780 or LSM 880 confocal laser-scanning microscope with a 20X lens or 43X water lens (Carl Zeiss, Oberkochen, Germany). In general, 8 to 20 layers with 1-μm thickness were scanned and stacked for each image unless otherwise stated, further processed, and presented as maximum intensity projection by the Fiji software (Schindelin et al., 2012).

### Cell Proliferation Analysis and TUNEL Staining

Embryos were first treated with 10 mM 5-Bromo-2′-deoxyuridine (BrdU, Sigma-Aldrich) pulses at the designated stage and then fixed with fresh 4% paraformaldehyde (PFA) in phosphate-buffered saline (PBS) to detect proliferating cells. We performed whole-mount IHC with mouse BrdU antibody (B2531, Sigma-Aldrich, 1:250) and detected apoptotic cells by TUNEL assay. Embryos were fixed with 4% PFA/PBS overnight at 4°C and dehydrated with methanol at −20°C. After gradual rehydration, the embryos were permeabilized with 10 μg/ml proteinase K for 2 min at room temperature, then post-fixed with 4% PFA/PBS. After several washes of PBS-T (0.1% tween-20 in PBS), embryos were again fixed with a pre-chilled solution containing ethanol and acetic acid (in 2:1 ratio) at −20°C for 10 min. We brought the pH value back with washes of PBS-T, and samples were incubated with 27 µl labeling solution plus 3 µl enzyme solution (*In Situ* Cell Death Detection Kit, AP, Roche) at 37°C overnight. They were washed three times with PBS-T for 5 min each and subjected to double whole-mount IHC staining with mouse anti-tubulin (acetyl Lys40) and rabbit anti-GFP antibodies.

### Time-Lapse Movies With a Light-Sheet Fluorescence Microscope

The time-lapse movies revealing the morphogenesis of neuromast regeneration were taken by the Zeiss Lightsheet Z. 1 (Carl Zeiss, Oberkochen, Germany). The other recordings showing macrophage dynamics were taken by a simple light sheet platform built by Dr. Bi-Chang Chen. This platform fascinatingly features 1) Bessel beam scanning, 2) dual illumination arms, 3) multiple software compatibility (μManager), 4) flexible objective combination, 4) large chamber as 12 mm × 12 mm × 25 mm, and 5) long-range XYZ motorized stage (MS-2000, Applied Scientific Instrumentation, United States).

### Tracking Analysis With Plugin “TrackMate”

To track the infiltration of macrophages during regeneration, we utilized a plugin, “TrackMate”, in the Fiji software (Tinevez et al., 2017). Since the z-sections focused were thin, we simplified the context by projecting the z-section with maximum intensity. We selected the LoG(Laplacian of Gaussian) detector with an estimated bulb diameter of 30 pixels to detect spots of interest. We used a simple LAP tracker with maximum linking distance (50 pixels), gap-closing maximum distance (15 pixels), and gap-closing maximum frame gap (3 frame gaps) to generate tracking paths. We manually corrected spots and links. We excluded spots not interacting with the pLL system or low resolution by filters (Y position and Quality). Spots are labeled with a radius ratio of 0.3–0.4, and tracks are presented with a color map set by the Track index.

### Nitroreductase/Metronidazole-Mediated Neuromast Ablation

Embryos from the cross of *Et(HG7L)* and *Tg(−8.0cldnb:NTR-hKikGR)* were collected and screened for double-positive ones. We freshly prepared a 10X stock of metronidazole (Mtz, M3761, SIGMA-ALDRICH) solution (20 mM Mtz, 1% DMSO). Larvae at three days post-fertilization (dpf) were first treated with a 1X working concentration of Mtz solution (2 mM Mtz, 0.1% DMSO diluted in 0.3% PTU containing 0.3X Danieau’s buffer) for 3, 6, 9, and 12 h in the dark. Then, the Mtz solution was replaced with several washes of fresh 0.3X Danieau’s buffer for recovery. The treatment of Mtz solution was 12 h to obtain the optimal ablation result.

### Pharmacological Inhibitors

All the chemical inhibitors were diluted in distilled water except for AG1478 [T4182, SIGMA-ALDRICH, in dimethyl sulfoxide (DMSO)] and LMT-28 (SML1628, SIGMA-ALDRICH, in methanol). Therefore, we used either 0.1% DMSO or 0.1% methanol as negative controls of AG1478 or LMT-28 treatments. AG1478 was added at 3 μM to the medium right after tail amputation. To disrupt different signaling pathways during Mtz ablation, we treated 3-dpf larvae with designated drugs and Mtz. After neuromast ablation, the chemical inhibitors were either washed out with Mtz or added back to be kept in the medium. The COX inhibitor diclofenac sodium salt (D6899, SIGMA-ALDRICH), a specific blocker of neutrophil recruitment, was used at 3 μM. The TNFα inhibitor pentoxifylline (PTX, P1784, SIGMA-ALDRICH) was used at 35 μM. The IL-6 inhibitors LMT-28 and the Wnt/β-catenin inhibitor IWR-1 (I0161, SIGMA-ALDRICH) were used at 10 μM.

### Liposome Injection for Macrophage Ablation

We used borosilicate glass capillaries (Sutter Instrument CO., B100-75-10, 1 mm O.D. X 0.75 mm I.D., Novato, CA, United States) to make injection needles by a Sutter puller (P-97, Sutter Instruments). We set the P-97 puller parameters for a shorter tip: air pressure 500, heat 510, pull 100, velocity 200, and time 60 (Benard et al., 2012). We then anesthetized 3-dpf larvae with 0.016% Tricaine/Ethyl 3-aminobenzoate methanesulfonate salt (A5040, SIGMA-ALDRICH). To ablate macrophages, we microinjected 5–8 nL of liposome-encapsulated clodronate (SKU# CLD-8909, Clodrosome) or control liposomes into their circulation system *via* the duct of Cuvier.

### Statistical Analysis

All experimental values except Figure 7 are presented as mean ± standard error and analyzed by one-way ANOVA. We indicate the total sample number in one experimental condition at the bottom or above the bar and label significantly different groups (*p* < 0.05) with different lettering. In Figure 7, raw data obtained from the TrackMate plugin, including spots, links, and tracks information, were further processed, analyzed, and presented as scatter plots or histograms by the “Ggplot2” package in the R software.

## Data Availability

The original contributions presented in the study are included in the article/Supplementary Materials; further inquiries can be directed to the corresponding author.

## Supplementary Material

Supplementary Figure S1 Generation and characterization of an enhancer-trapped line, *Et(HG7L)*, expressing EGFP in interneuromast and mantle cells. We generated an enhancer trap line *Et(HG7L)* line by random insertions of a Tol2 construct containing a heat-shock 70 (hsp70) promoter and an EGFP gene. The insertion site was between *ccdc147* and *sorcs3* in Chromosome 1 by inverse PCR analysis **(A)**. We examined a larva two days post-fertilization (dpf) under epifluorescence microscopy, photographed anterior to the left on the lateral side **(B)**. Magnified dorsal and lateral views of the anterior head and posterior region **(D)** are shown in **(C, D)**, respectively. We fixed the larvae and subjected them to whole-mount in situ hybridization against *sorcs3* (**E**, the anterior head region in dorsal view) or *ccdc147* (**F**, the posterior tail region in lateral view). (**G–L**) Larvae at 3-dpf from the cross of *Et(HG7L)* and *Tg(-4.7alpl:mCherry)* were immobilized and examined under confocal microscopy at EGFP (green) and mCherry (red) channel. At the EGFP channel, the *Et(HG7L)* revealed EGFP in interneuromast cells (INCs) and mantle cells (MCs) and also a subpopulation of supporting cells showing weaker expression **(G, H)**. At the mCherry channel, the *Tg(-4.7alpl:mCherry)* had mCherry in INCs and MCs but not supporting cells **(I, J)**. The superimposed green and red channel images are shown below **(K, L)**. **(M)** A graph depicts the expressions of EGFP (green) and mCherry (red) in the *Et(HG7L)* (left) and *Tg(-4.7alpl:mCherry)* (right) lines, respectively.

Supplementary Figure S2 A rosette forms within the regenerating cluster as a landmark of neuromast formation. **(A)** We used larvae from the cross of *Tg(−8.0cldnb:lyn-GFP)* *(cldnb:lynGFP in green)* and *Tg(−4.7alpl:mCherry)* *(alpl:mCherry in red)* to reveal morphogenesis of neuromast regeneration post-fin amputation. The cldnb:lynGFP line expressed green EGFP in all cell types of the lateral line, and the alpl:mCherry line expressed red mCherry in interneuromast cells and mantle cells, as shown in the cartoon. **(B)** At two days post-amputation (dpa), the double transgenic larvae had formed a cluster (boxed with a dashed line) as examined under confocal microscopy. **(C)** The regenerating cluster was scanned at higher magnification for 10 1.76-um stacks labeled z1-10. Stacks z4-6 are shown, and a rosette (arrowhead) was seen in the z-4 and z-5 stacks. **(D)** Larvae were also fixed and subjected to immunohistochemistry against ZO-1 expressed in tight junctions (red) and GFP expressed in lateral lines (green). A strong ZO-1 signal was observed in the cluster, as indicated by an arrow indicating polarity establishment. **(E)** To compare clusters with and without a rosette, we measured the area, length, and width of a cluster as shown in a cartoon on the left (a yellow asterisk represents a rosette) and calculated their area and length/width ratio. The “X” indicates the distance between the rosette center and the lagging end of the cluster. Scatter plots are presented, and each dot represents the measurement from one larva. Mean ± s.e.m. are shown. Data were analyzed by one-way ANOVA (n = 18).

Supplementary Figure S3 Characterization of a *Tg(-8.0cldnb:NTR-hKikGR;myl7:EGFP)* double-transgenic zebrafish line. **(A)** The design of a transgenic cassette composed of an 8-kb claudin b promoter (−8.0 cldnb), a nitroreductase gene (NTR) fused with a hKikGR a myl7 promoter, and an EGFP gene. The transgenic cassette is expected to express green fluorescence along the lateral line and the missing heart (as depicted in a cartoon on the right. **(B)** The *Tg(-8.0cldnb:NTR-hKikGR;myl7:EGFP)* larvae at three days post-fertilization (dpf) were fixed and examined under confocal microscopy GFP channel. It showed a myl7-driven solid green fluorescence in the heart (white asterisk) that served as a convenient selection marker. Punctate green fluorescence was found in neuromasts, as demonstrated in a representative neuromast enclosed by a dashed rectangle along anterior (head region to the left) and posterior lateral line systems (trunk region to the right). A superimposed dark and bright field image are shown below. **(C)** Larvae from the cross of *Tg(-8.0cldnb:NTR-hKikGR;myl7:EGFP)* and *Et(HG7L)* were fixed, incubated with DAPI, examined, and photographed at the GFP and UV channel. Representative superimposed images for a neuromast of 3 and 4-dpf larvae are presented. Punctate green fluorescence from NTR-hKikGR proteins was found around blue nuclei stained by DAPI in every cell within a neuromast.

Supplementary Figure S4 Neutrophil recruitment was impaired by diclofenac sodium post-amputation. **(A)** We used larvae from a transgenic line, *Tg(mpx:GFP)*, expressing GFP in neutrophils, and clipped their tail fins three days post-fertilization (dpf). After amputation, larvae were transferred to a dish without or with Diclofenac. We counted the number of neutrophils recruited to the proximal amputated tail at designated stages (*N* = 3, n = 25 for the control group, n = 24 for the Diclofenac 3 μM group, n = 27 for the Diclofenac 6 μM group). **(B)** Mtz-treated larvae from the cross of *Tg(−8.0cldnb:NTR-hKikGR;Et(HG7L))* and *Tg(mpeg1:mCherry;mpx:GFP)* were also transferred to a dish without or with 3 μM Diclofenac and the number of neutrophils recruited to the NTR-ablated neuromasts were counted at designated stages (*N* = 3, n = 27 for the control group, n = 31 for the Diclofenac group).

Supplementary Figure S5 Macrophage ablation retards the regeneration of neuromast post tail amputation. **(A)** At 3 days post-fertilization, *Et(HG7L)* larvae were fin-amputated and cultured in a designated diclofenac sodium concentration. We counted the number of survived larvae at each phase and treatment at the designated stages, and the percentages of larvae are shown (N = 4). **(B)** The vehicle control, encapsome (En), or clodronate liposomes (Clo) were injected into the posterior cardinal vein (PCV) of an Et(HG7L) larvae 3 days post-fertilization, and the fin was amputated after injection. The surviving larvae at each phase and treatment were counted at the designated day post-amputation (dpa), and the percentages of larvae are shown (*N* = 3).

Supplementary Figure S6 Macrophages display distinct cell behaviors, including “Stay,” “Patrol” and “Flash” during neuromast regeneration. **(A–C)** Cartoons show macrophages (red) migrate along INCs (bright green with borderline) and underneath SWCs (light green without borderline) with differential directions and different velocities. We thus categorized these behaviors as **(A)** “Stay” with minimal movements, **(B)** “Patrol” with limited movements and frequent changes in direction, and **(C)** “Flash” with swift unidirectional movement. **(D)** We cropped snapshots of a 70-min movie from Supplementary Video S5. An arrow, arrowhead, or asterisk indicates a “Stay,” “Patrol” or “Flash” macrophage, respectively, in each frame. Please note the migrating distance and direction of a macrophage along the X-axis to appreciate the difference between different types of behavior.

Supplementary Figure S7 During development, macrophages interact with 2nd posterior lateral line primordium (2nd pLLp) migration. We imaged larvae from the cross of *Tg(mpeg1:mCherry;FoxD3:GFP)* and *Tg(−8.0cldnb:NTR-hKikGR;Et(HG7L))* at 4 days post-fertilization (hpf) under confocal microscopy. Snapshots shown are representative confocal stacked images in the orthogonal views, while two X-Z views and one Y-Z view are shown below and on the right side, respectively. Solid or dashed horizontal lines indicated two separate X-Z views (upper or lower). During development, the 2nd pLLp migrates from the otic vesicle at later stages, along the same path as the 1st pLLp. The 2nd pLLp would separate SWCs (white asterisks) and INCs, leading to intercalary formation. We found macrophages (red) indicated by arrowheads between the 2nd pLLp and INCs or SWCs. Scale bars are the same as 10 μm.

Supplementary Video S1 The tail fin of an *Et(HG7L)* larva at 3 days post-fertilization (3 dpf) was cut to remove neuromast L6-8 as described in Figure 1B, embedded in agar and imaged under confocal microscopy. Images were taken at 6 min per frame for 6 h. This video shows the Phase I of neuromast regeneration described in Figure 1C. At the leading end toward the cutting edge (bottom), most interneuromast cells (INCs) stayed in line with visible cell protrusions (arrowheads). Several INCs (arrows) were seen to crawl onto the original INC. Time is shown in the top right corner.

Supplementary Video S2 The *Et(HG7L)* larva was treated as described in Supplementary Video 1. This video shows Phase II of neuromast regeneration described in Figure 1C. A large cluster was formed with many visible cell protrusions (arrowheads) at both the leading and lagging end toward the cutting edge at the bottom.

Supplementary Video S3 The *Et(HG7L)* larva was treated as described in Video 1. This video shows the Phase III of neuromast regeneration described in Figure 1C. A new neuromast was formed with a clear ring-like structure and GFP-labeled mantle cells.

Supplementary Video S4 AG1478 caused hyperactive cell protrusion and cell division. Cell protrusions are marked by arrowheads. White and magenta asterisks, respectively, label the mother cell and two daughter cells.

Supplementary Video S5
*In toto* imaging analysis shows differential macrophage (red) behaviors during regeneration. Larvae from the cross of *Tg(mpeg1:mCherry;FoxD3:GFP)* and *Tg(−8.0cldnb:NTR-hKikGR) Et(HG7L)* were treated with Mtz and monitored under a light-sheet fluorescent microscope. Time-lapse movies of merged channels with cell tracking (purple circles, middle row) are presented. Images were taken at 5 min per frame for 6 h 35 min, as shown on the top left.

Supplementary Video S6 Uneven distribution of recruited macrophages (red) during regeneration was analyzed and presented in the bar chart (upper row) graphic interchange format to reveal the dynamics of macrophage distribution. Larvae from the cross of *Tg(mpeg1:mCherry;FoxD3:GFP)* and *Tg(-8.0cldnb:NTR-hKikGR);Et(HG7L)* were treated with Mtz and observed under a light-sheet fluorescence microscope. Time-lapse movies of merged channels with cell tracking (middle row), and red channel with cell tracking (bottom row) are combined vertically and shown here. Images were taken at 5 min per frame for 11 h 55 min as shown on the top left of images.

Supplementary Video S7 This video is a three-dimensional pseudocolor reconstruction. While most macrophages (red, left) were crawling on INCs (left), some macrophages (red, middle) could scroll through the space between INCs and underneath SWCs (right).

Supplementary Video S8 Macrophages (red) were not only in contact with interneuromasts (green, arrow) but were also dynamically embracing the second posterior lateral line primordium (green, open arrow) during development.

## Abbreviations

INCs, interneumast cells; SCs, supporting cells; HCs, hair cells; MCs, mantle cells; SWCs, Schwann cells; dpf, days post-fertilization; dpa, days post-amputation.
