# Supplementary material for: Macrophages Break Interneuromast Cell Quiescence by Intervening in the Inhibition of Schwann Cells in the Zebrafish Lateral Line
Source: Front Cell Dev Biol. 2022 Jul 1;10:907863. doi: 10.3389/fcell.2022.907863 (PMC9285731; doi:10.3389/fcell.2022.907863)
Supplement: Supplementary file 3 [file Presentation1.pdf]

SUPPLEMENTARY FIGURE & LEGENDS

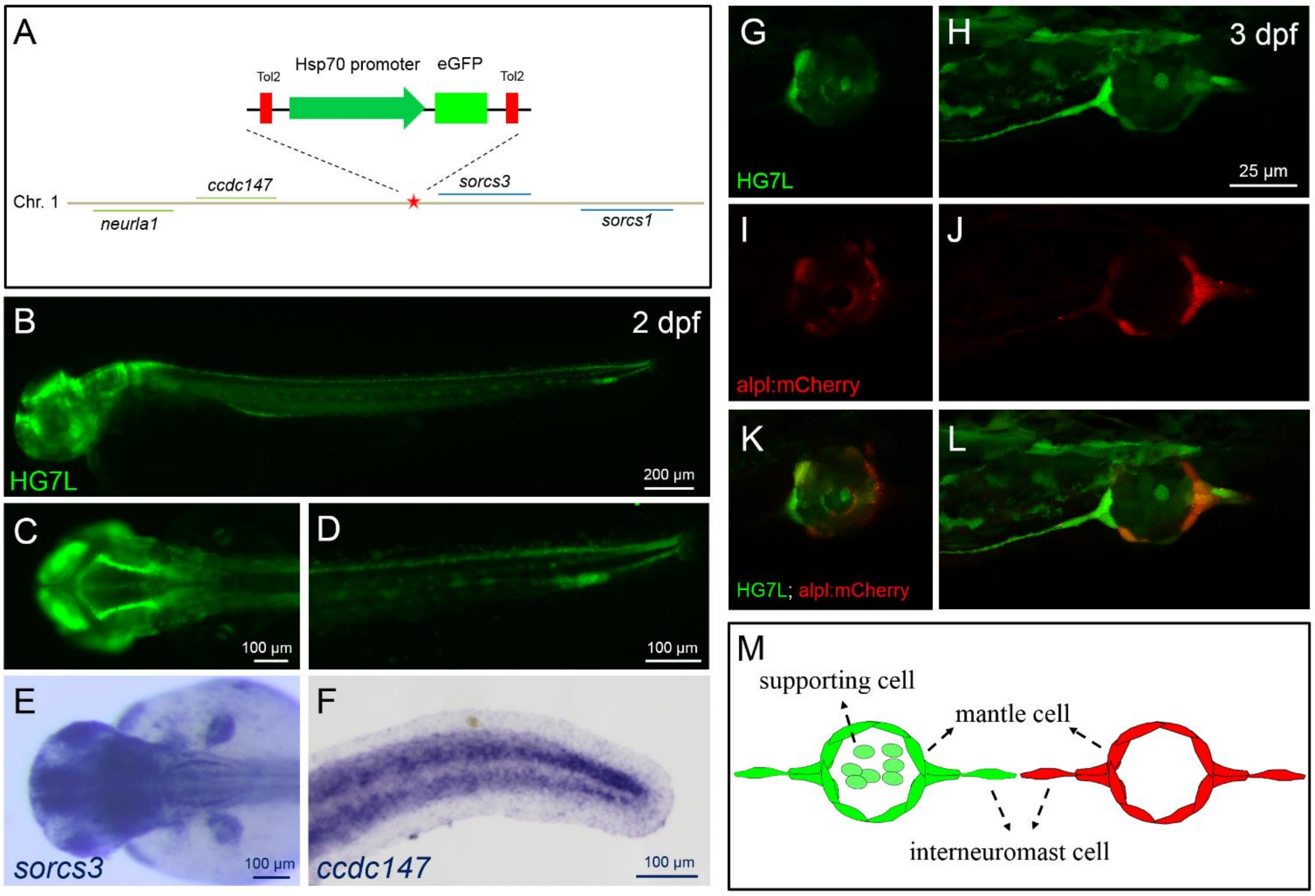

Figure S1. *Generation and characterization of an enhancer-trapped line, Et(HG7L), expressing EGFP in interneuromast and mantle cells.* We generated an enhancer trap line *Et(HG7L)* line by random insertions of a Tol2 construct containing a heat-shock 70 (hsp70) promoter and an EGFP gene. The insertion site was between *ccdc147* and *sorcs3* in Chromosome 1 by inverse PCR analysis (A). We examined a larva two days post-fertilization (dpf) under epifluorescence microscopy, photographed anterior to the left on the lateral side (B). Magnified dorsal and lateral views of the anterior head and posterior region (D) are shown in (C) and (D), respectively. We fixed the larvae and subjected them to whole-mount *in situ* hybridization against *sorcs3* (E, the anterior head region in dorsal view) or *ccdc147* (F, the posterior tail region in lateral view). (G-L) Larvae at 3-dpf from the cross of *Et(HG7L)* and *Tg(-4.7alpl:mCherry)* were immobilized and examined under confocal microscopy at EGFP (green) and mCherry (red) channel. At the EGFP channel, the *Et(HG7L)* revealed EGFP in interneuromast cells (INCs) and mantle cells (MCs) and also a subpopulation of supporting cells showing weaker expression (G, H). At the mCherry channel, the *Tg(-4.7alpl:mCherry)* had mCherry in INCs and MCs but not supporting cells (I, J). The superimposed green and red channel images are shown below (K, L). (M) A graph depicts the expressions of EGFP (green) and mCherry (red) in the *Et(HG7L)* (left) and *Tg(-4.7alpl:mCherry)* (right) lines, respectively.

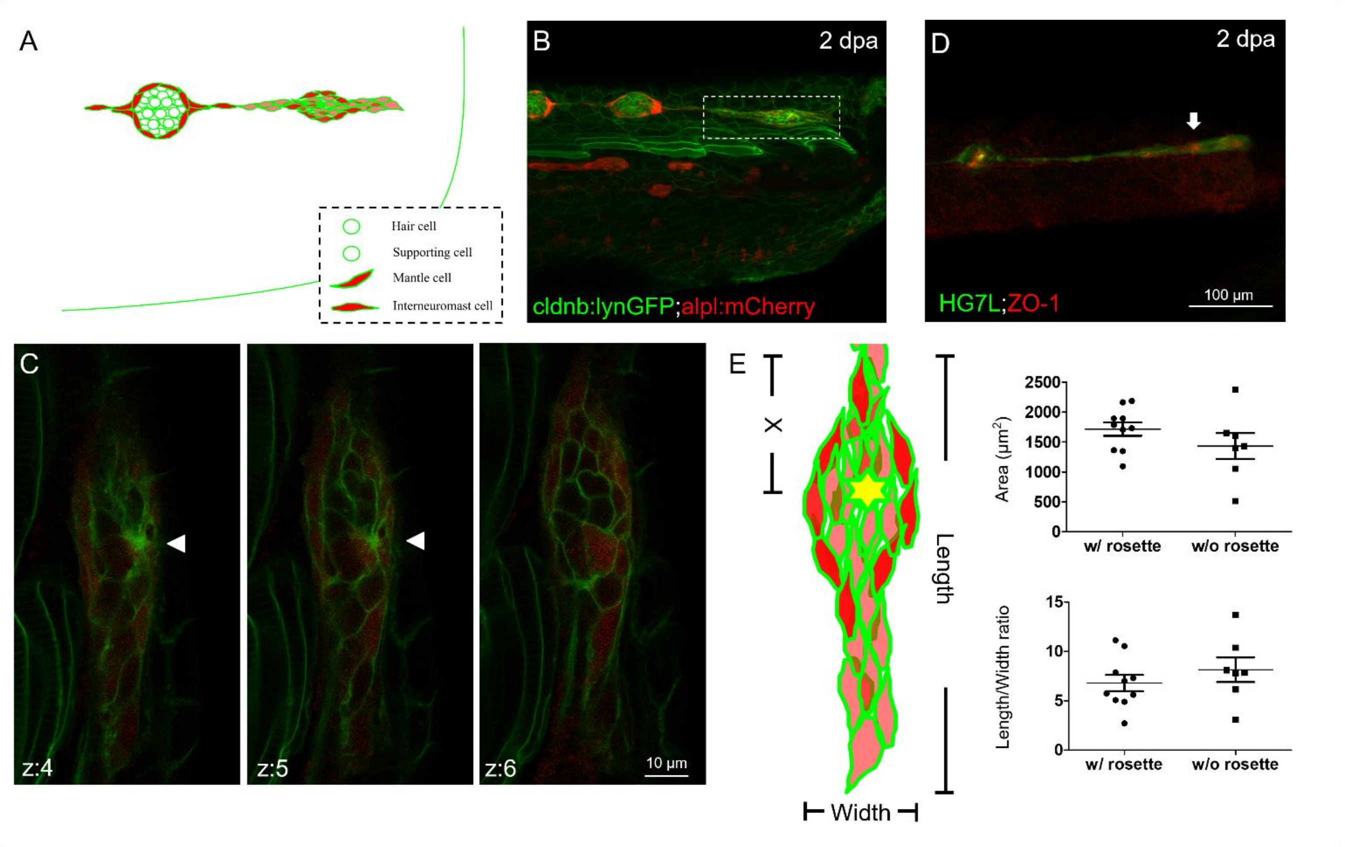

Figure S2. *A rosette forms within the regenerating cluster as a landmark of neuromast formation.* (A) We used larvae from the cross of *Tg(-8.0cldnb:lyn-GFP)* (cldnb:lynGFP in green) and *Tg(-4.7alpl:mCherry)* (alpl:mCherry in red) to reveal morphogenesis of neuromast regeneration post-fin amputation. The cldnb:lynGFP line expressed green EGFP in all cell types of the lateral line, and the alpl:mCherry line expressed red mCherry in interneuromast cells and mantle cells, as shown in the cartoon. (B) At two days post-amputation (dpa), the double transgenic larvae had formed a cluster (boxed with a dashed line) as examined under confocal microscopy. (C) The regenerating cluster was scanned at higher magnification for 10 1.76-um stacks labeled z1-10. Stacks z4-6 are shown, and a rosette (arrowhead) was seen in the z-4 and z-5 stacks. (D) Larvae were also fixed and subjected to immunohistochemistry against ZO-1 expressed in tight junctions (red) and GFP expressed in lateral lines (green). A strong ZO-1 signal was observed in the cluster, as indicated by an arrow indicating polarity establishment. (E) To compare clusters with and without a rosette, we measured the area, length, and width of a cluster as shown in a cartoon on the left (a yellow asterisk represents a rosette) and calculated their area and length/width ratio. The “X” indicates the distance between the rosette center and the lagging end of the cluster. Scatter plots are presented, and each dot represents the measurement from one larva. Mean ± s.e.m. are shown. Data were analyzed by one-way ANOVA (n = 18).

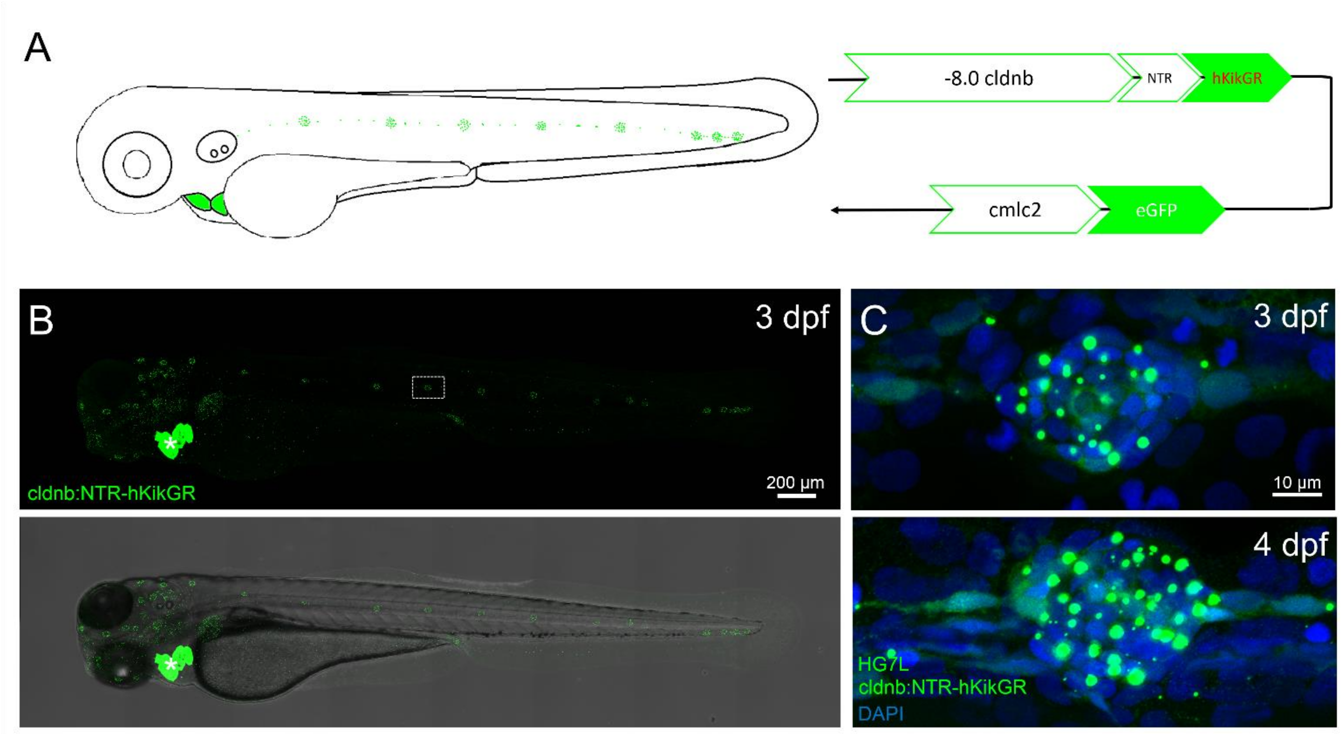

Figure S3. *Characterization of a Tg(-8.0cldnb:NTR-hKikGR;myl7:EGFP) double-transgenic zebrafish line.* (A) The design of a transgenic cassette composed of an 8-kb claudin b promoter (-8.0 cldnb), a nitroreductase gene (NTR) fused with a hKikGR a *myl7* promoter, and an EGFP gene. The transgenic cassette is expected to express green fluorescence along the lateral line and the missing heart (as depicted in a cartoon on the right. (B) The *Tg(-8.0cldnb:NTR-hKikGR;myl7:EGFP)* larvae at three days post-fertilization (dpf) were fixed and examined under confocal microscopy GFP channel. It showed a myl7-driven solid green fluorescence in the heart (white asterisk) that served as a convenient selection marker. Punctate green fluorescence was found in neuromasts, as demonstrated in a representative neuromast enclosed by a dashed rectangle along anterior (head region to the left) and posterior lateral line systems (trunk region to the right). A superimposed dark and bright field image are shown below. (C) Larvae from the cross of *Tg(-8.0cldnb:NTR-hKikGR;myl7:EGFP)* and *Et(HG7L)* were fixed, incubated with DAPI, examined, and photographed at the GFP and UV channel. Representative superimposed images for a neuromast of 3 and 4-dpf larvae are presented. Punctate green fluorescence from NTR-hKikGR proteins was found around blue nuclei stained by DAPI in every cell within a neuromast.

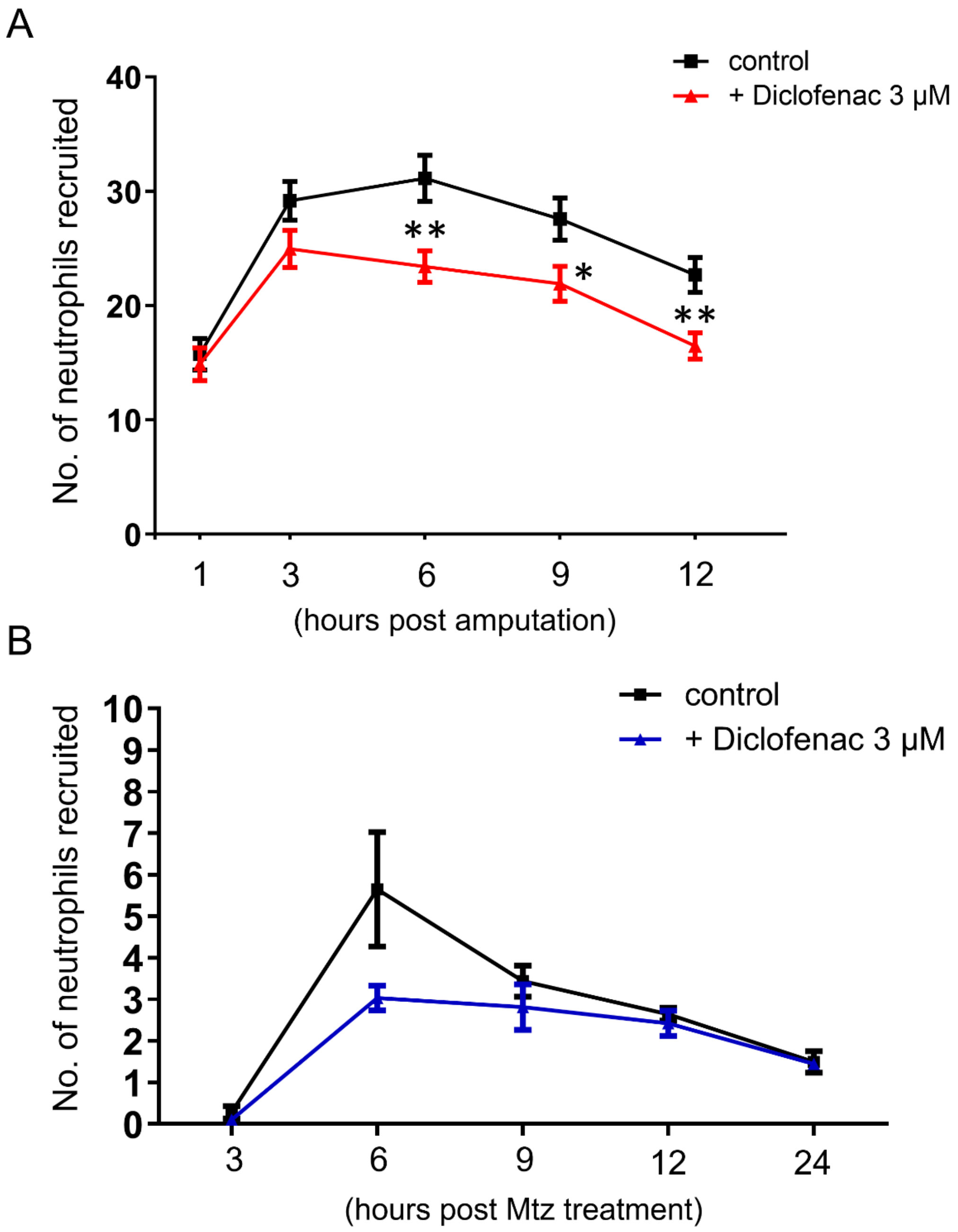

Figure S4. *Neutrophil recruitment was impaired by diclofenac sodium post-amputation.* (A) We used larvae from a transgenic line, *Tg(mpx:GFP)*, expressing GFP in neutrophils, and clipped their tail fins three days post-fertilization (dpf). After amputation, larvae were transferred to a dish without or with Diclofenac. We counted the number of neutrophils recruited to the proximal amputated tail at designated stages (N = 3, n = 25 for the control group, n = 24 for the Diclofenac 3 μM group, n = 27 for the Diclofenac 6 μM group). (B) Mtz-treated larvae from the cross of *Tg(-8.0cldnb:NTR-hKikGR;Et(HG7L))* and *Tg(mpeg1:mCherry;mpx:GFP)* were also transferred to a dish without or with 3 μM Diclofenac and the number of neutrophils recruited to the NTR-ablated neuromasts were counted at designated stages (N = 3, n = 27 for the control group, n = 31 for the Diclofenac group).

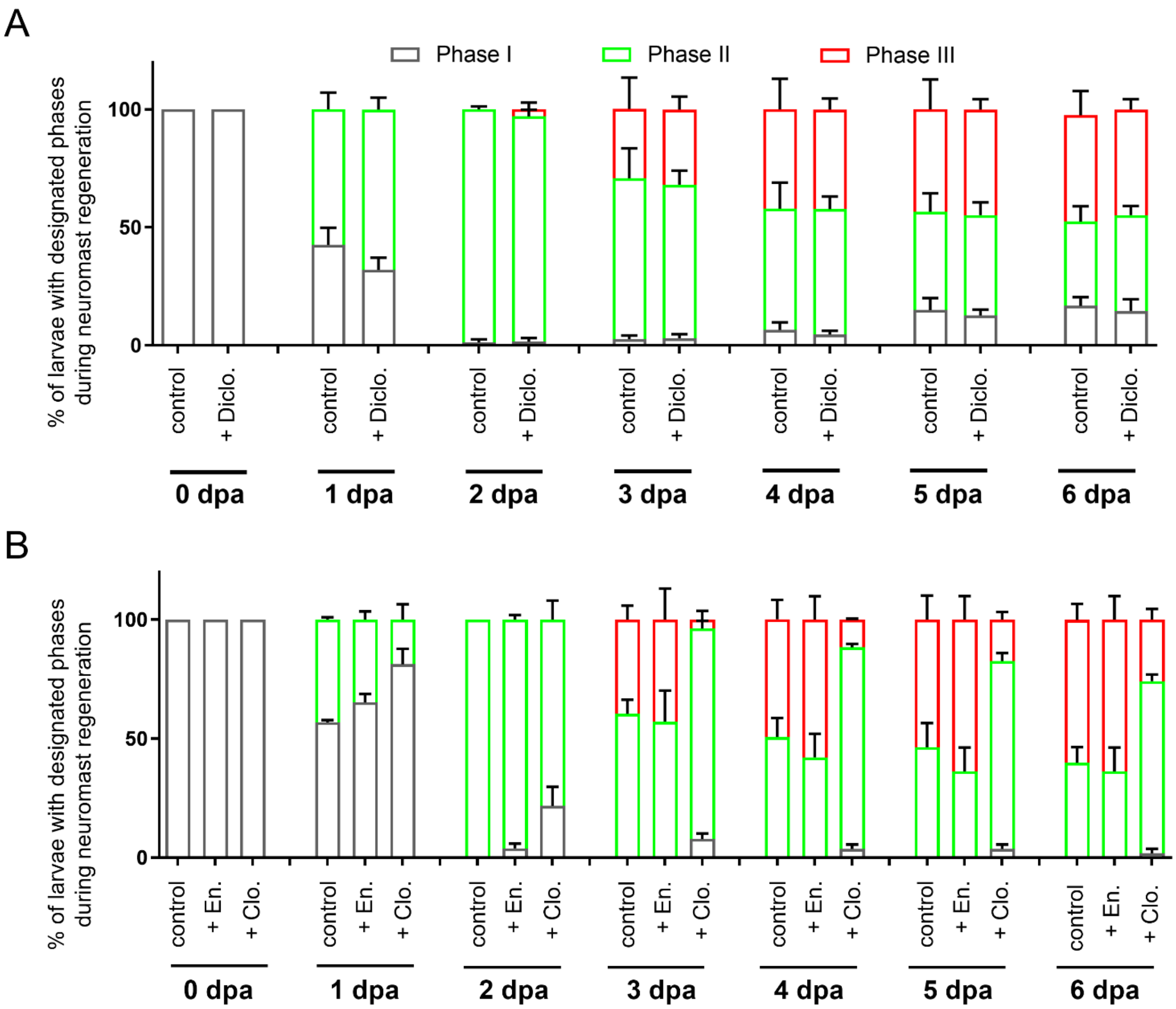

Figure S5. *Macrophage ablation retards the regeneration of neuromast post tail amputation.* (A) *At three days post-fertilization, Et(HG7L) larvae* were fin-amputated and cultured in a designated diclofenac sodium concentration. We counted the number of survived larvae at each phase and treatment at the designated stages, and the percentages of larvae are shown (N = 4). (B) The vehicle control, encapsome (En), or clodronate liposomes (Clo) were injected into the posterior cardinal vein (PCV) of an *Et(HG7L)* larvae three days post-fertilization, and the fin was amputated after injection. The surviving larvae at each phase and treatment were counted at the designated day post-amputation (dpa), and the percentages of larvae are shown (N = 3).

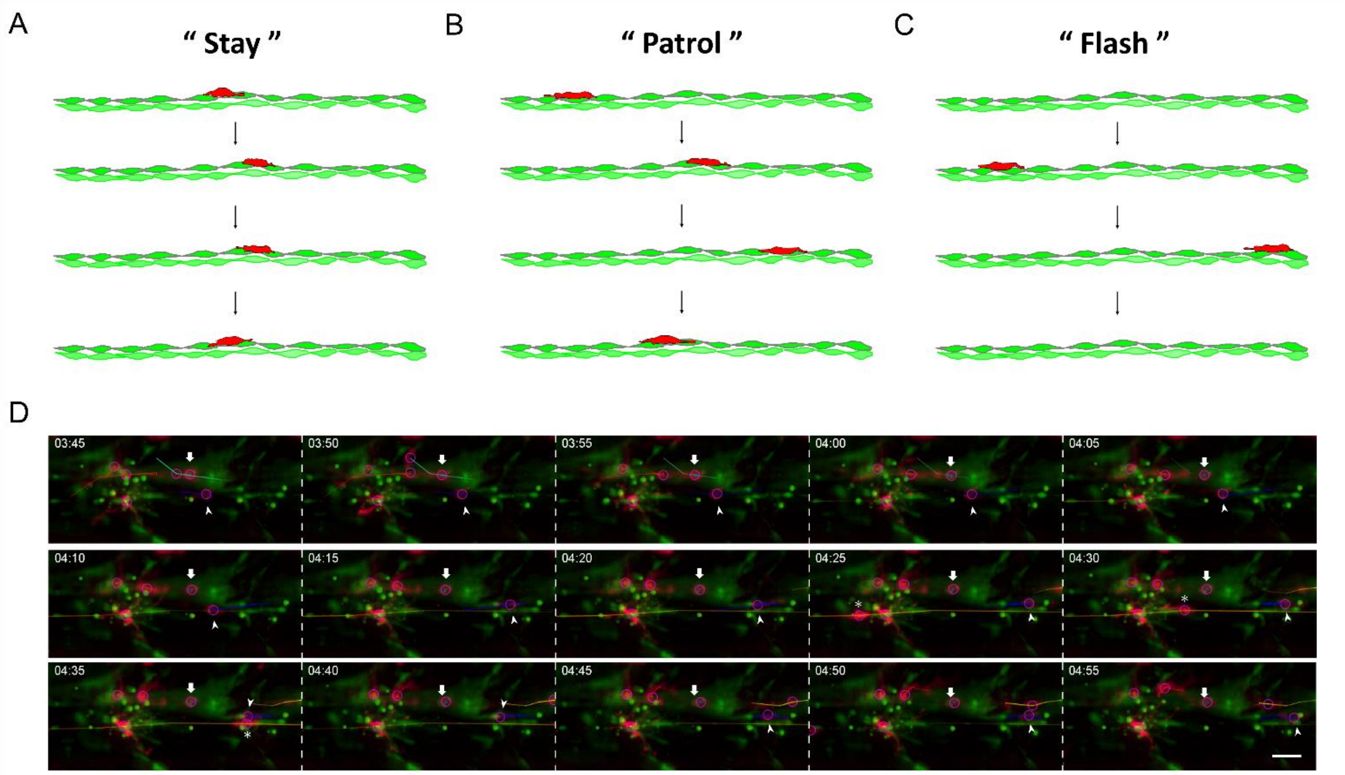

Figure S6. *Macrophages display distinct cell behaviors, including "Stay", "Patrol" and "Flash" during neuromast regeneration.* (A-C) Cartoons show macrophages (red) migrate along INCs (bright green with borderline) and underneath SWCs (light green without borderline) with differential directions and different velocities. We thus categorized these behaviors as (A) "Stay" with minimal movements, (B) "Patrol" with limited movements and frequent changes in direction, and (C) "Flash" with swift unidirectional movement. (D) We cropped snapshots of a 70-min movie from supplementary Video 5. An arrow, arrowhead, or asterisk indicates a "Stay", "Patrol" or "Flash" macrophage, respectively, in each frame. Please note the migrating distance and direction of a macrophage along the X-axis to appreciate the difference between different types of behavior.

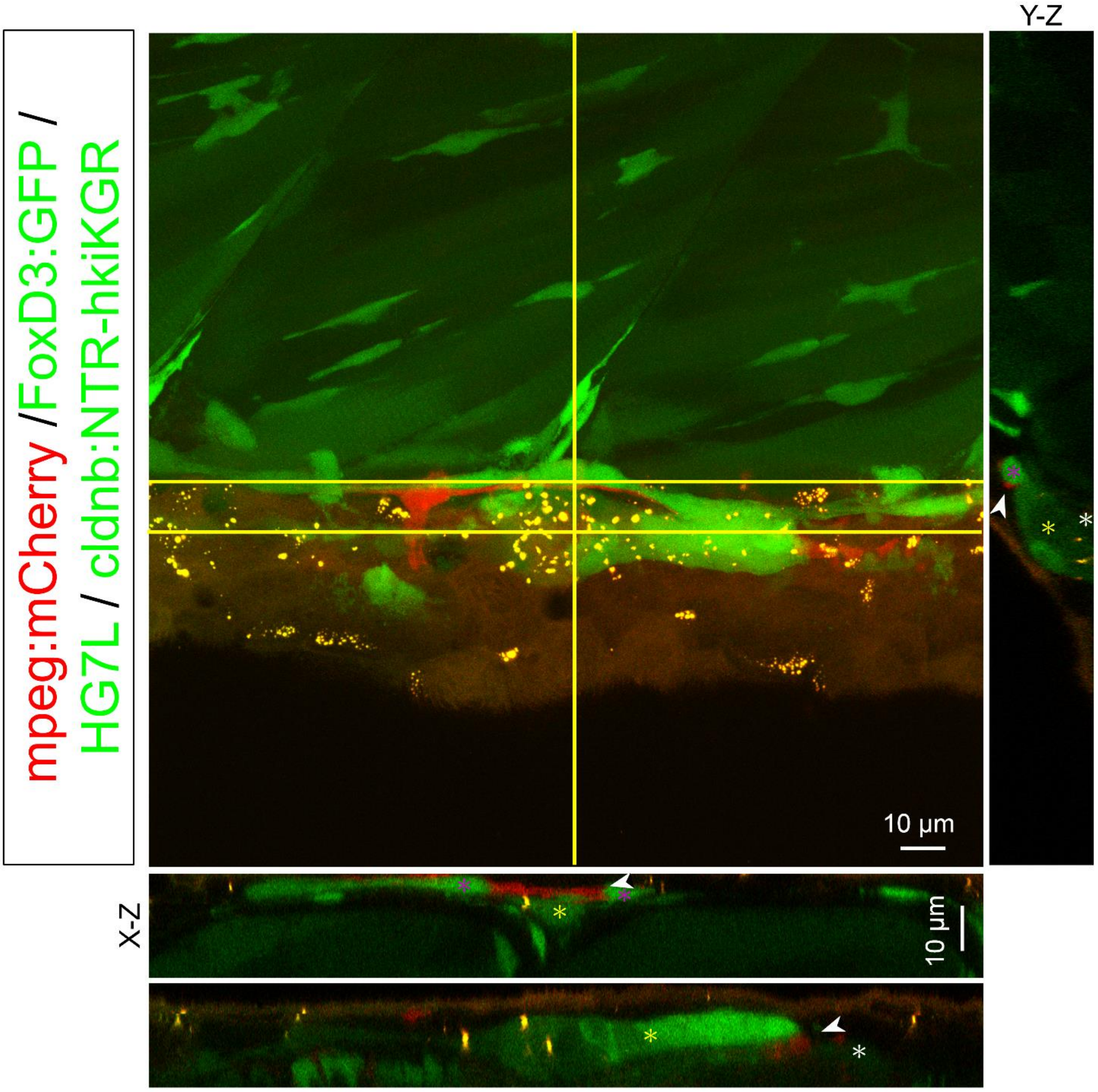

Figure S7. *During development, macrophages interact with 2nd posterior lateral line primordium (2nd pLLp) migration.* We imaged larvae from the cross of *Tg(mpeg1:mCherry;FoxD3:GFP)* and *Tg(-8.0cldnb:NTR-hKikGR;Et(HG7L))* at four days post-fertilization (hpf) under confocal microscopy. Snapshots shown are representative confocal stacked images in the orthogonal views, while two X-Z views and one Y-Z view are shown below and on the right side, respectively. Solid or dashed horizontal lines indicated two separate X-Z views (upper or lower). During development, the 2nd pLLp migrates from the otic vesicle at later stages, along the same path as the 1st pLLp. The 2nd pLLp would separate SWCs (white asterisks) and INCs, leading to intercalary formation. We found macrophages (red) indicated by arrowheads between the 2nd pLLp and INCs or SWCs. Scale bars are the same as 10 μm.
